# Supplementary material for: Sex differences in sexual attraction for aesthetics, resources and personality across age
Source: PLoS One. 2021 May 19;16(5):e0250151. doi: 10.1371/journal.pone.0250151 (PMC8133465; doi:10.1371/journal.pone.0250151)
Supplement: S1 Appendix — (DOCX) [file pone.0250151.s001.docx]

**S1 Appendix**


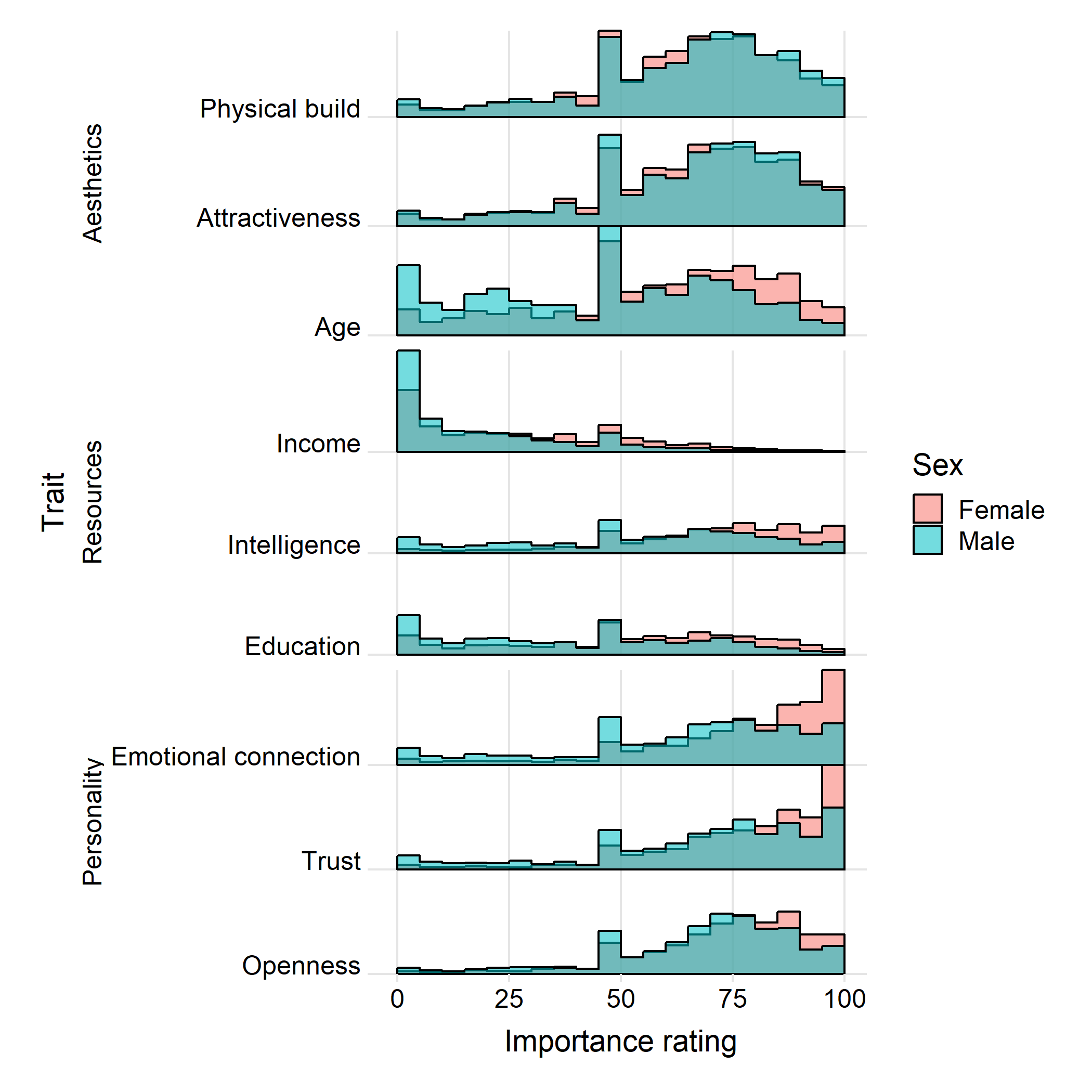


**A1 Fig.** Distribution of the characteristics’ importance rating for sexual attraction, by respondents’ sex.

*Note:* Bin width of 5. *N_males_*= 4,375; *N_females_* = 2,685.

**
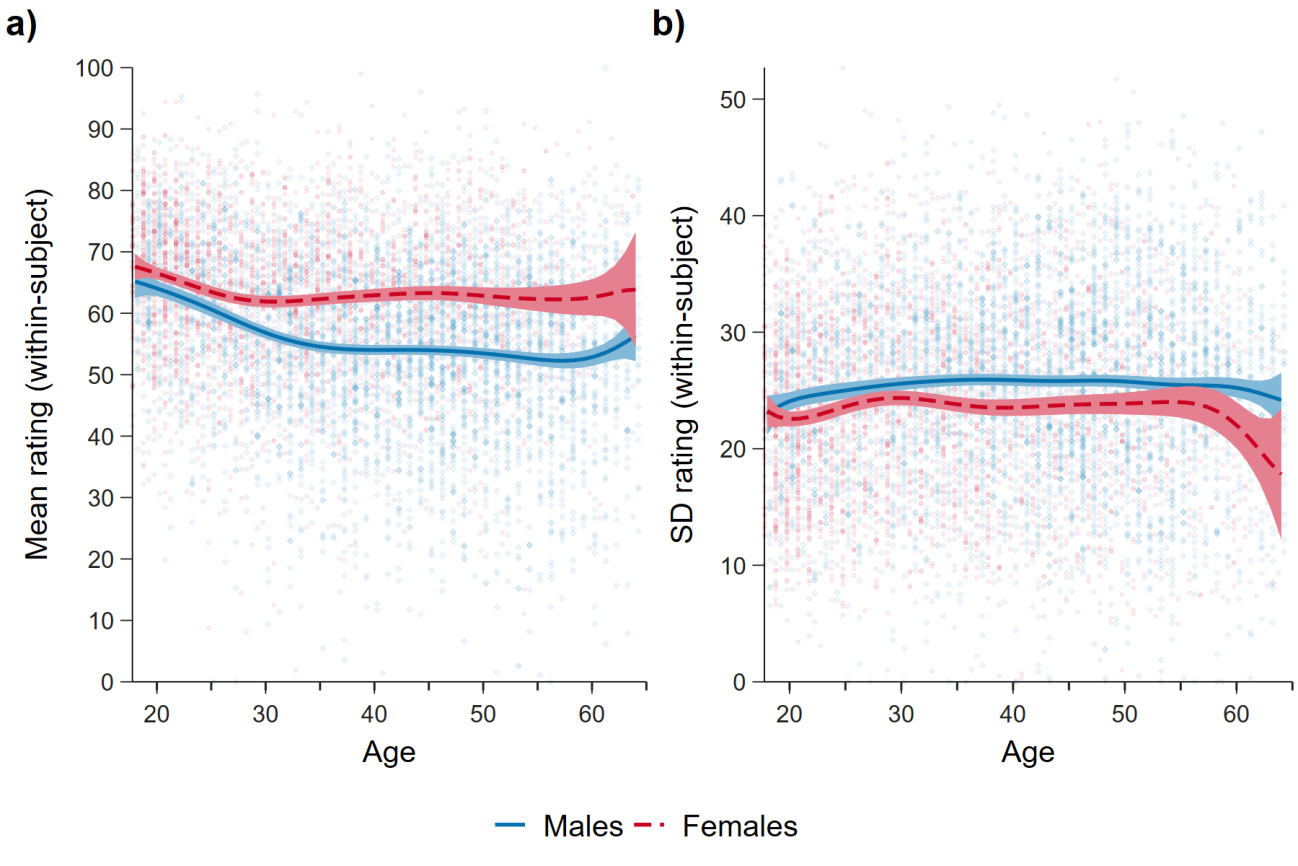
**

**A2 Fig.** Within-subject rating average (a) and standard deviation (b) over age, by sex.

*Notes*: *N_males_*= 4,375; *N_females_* = 2,685. Smoothed local cubic polynomials (Gaussian kernel function with the bandwidth 5) with 95% confidence intervals (shaded areas). Markers represent individual observations. For better visualization, markers are offset by +-0.25 for females (red) and males (blue), respectively.


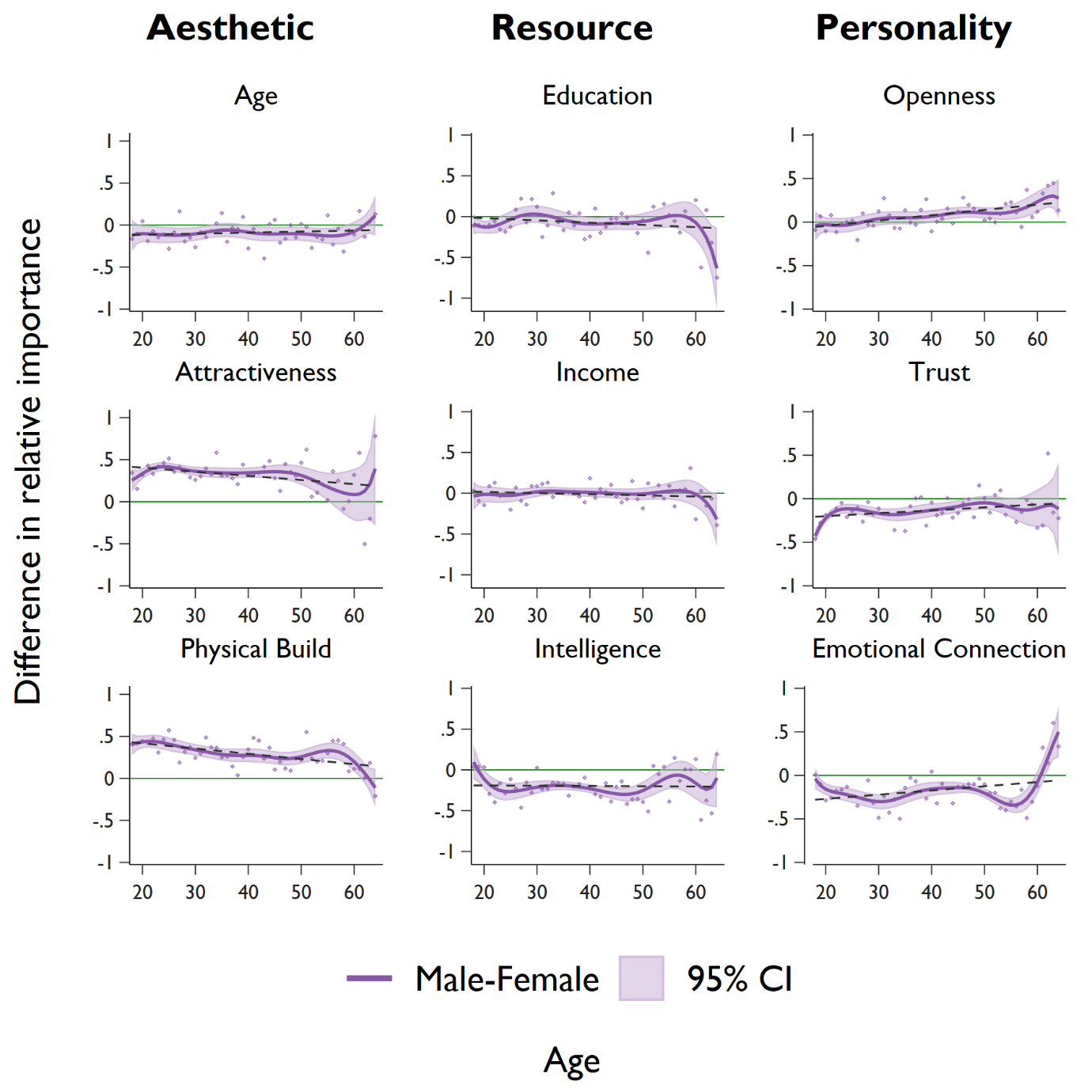


**A3 Fig.** Sex differences in relative importance to sexual attraction across age.

*Notes*: Sex differences are calculated by taking the males’ average relative importance at each year of age and subtracting it from the females’ average relative importance at the same age; outcomes represented by the markers. Purple lines show the smoothed local cubic polynomials (Gaussian kernel function with the bandwidth 5) with 95% confidence intervals (shaded areas).


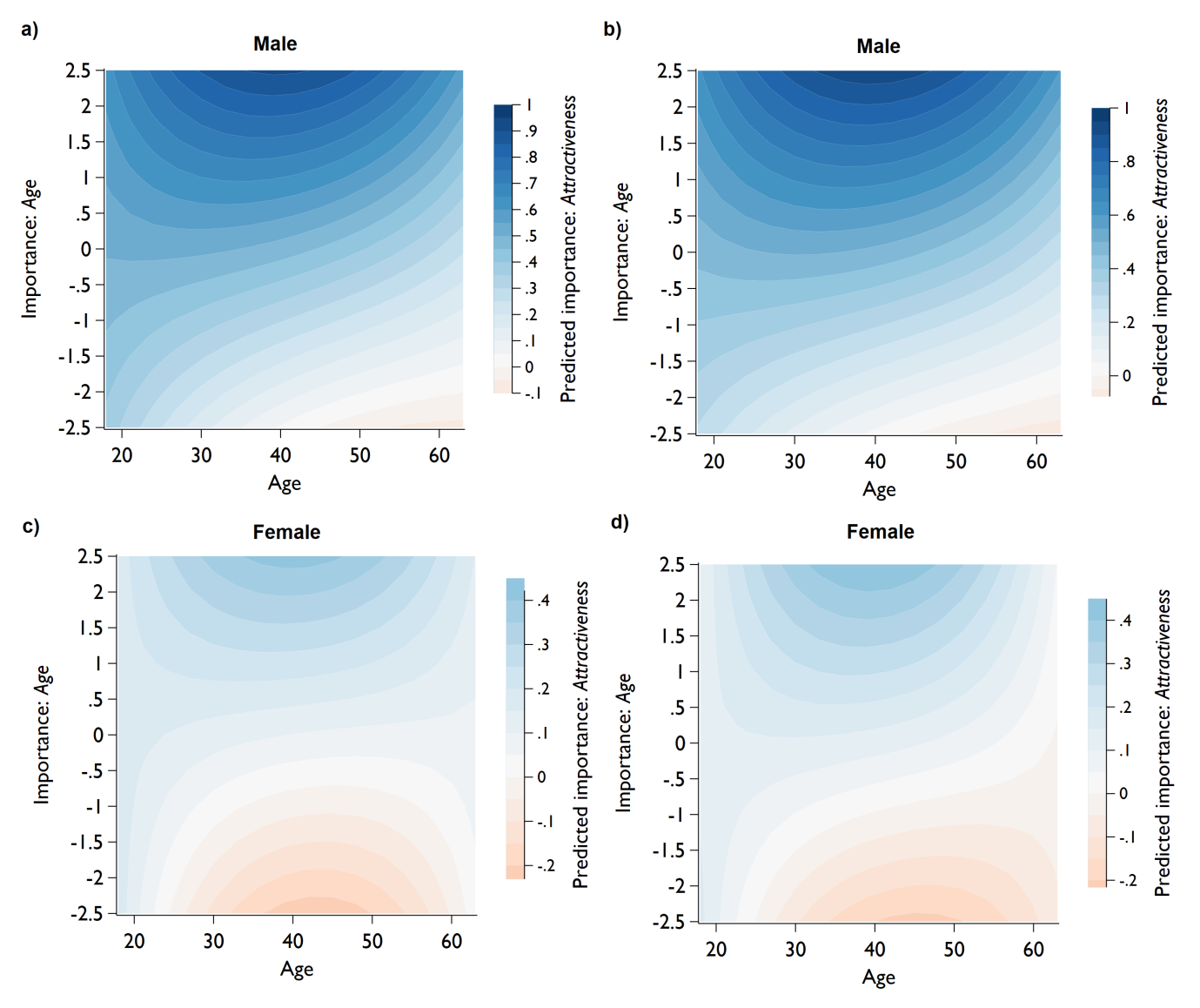


**A4 Fig.** Predicted importance of *attractiveness* for sexual attraction across male’s (panel **a** and **b**) and female’s (panel **c** and **d**) age, mediated by relative importance of *age*.

*Note:* Predicted importance of attractiveness (represented by color) were obtained from OLS regressions with the interaction term of importance of age (y-axis) and age and age square (x-axis). Control variables were omitted in panel **a** and **c** and included in panel **b** and **d**.


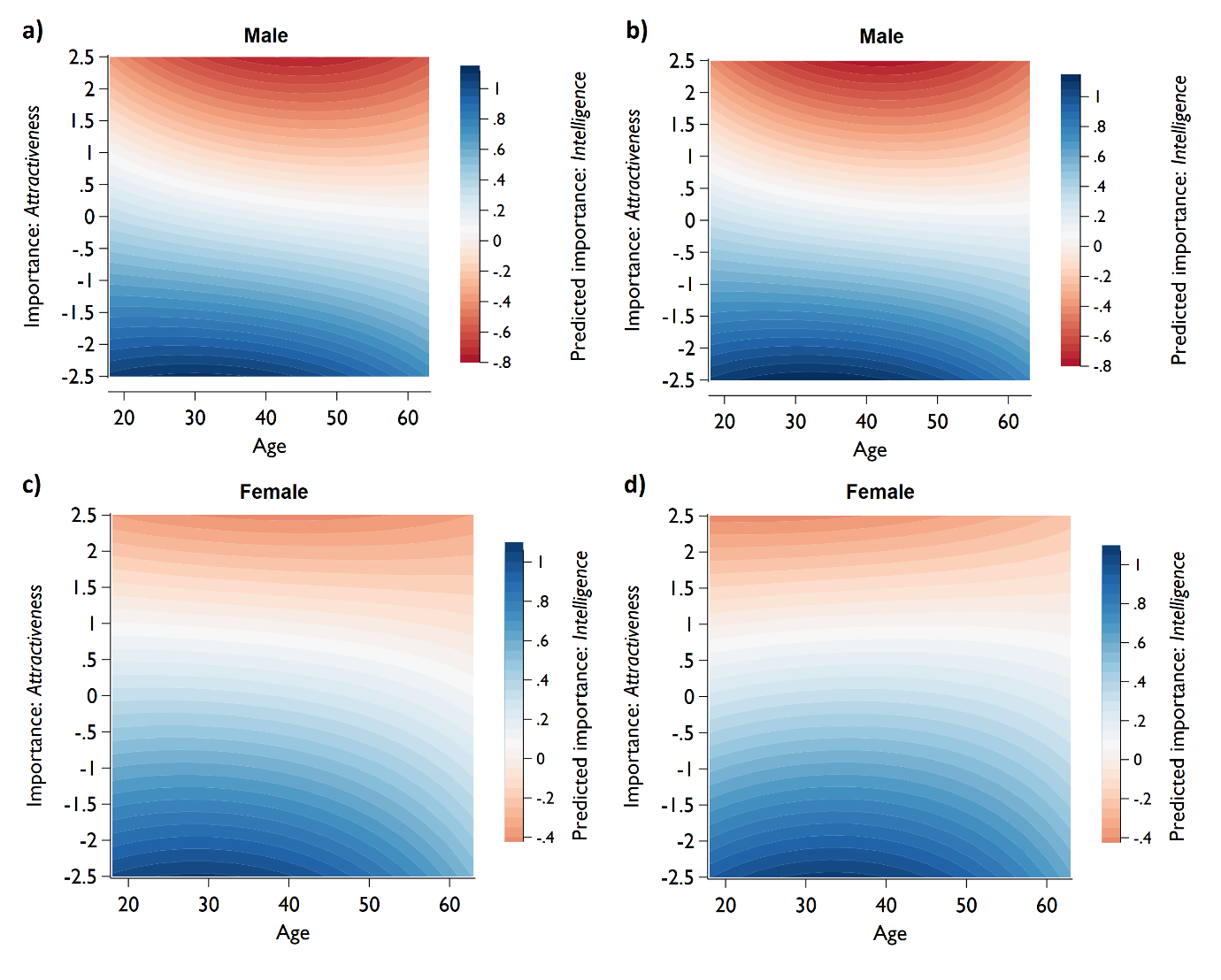


**A5 Fig.** Predicted importance of *intelligence* for sexual attraction across male’s (panel **a** and **b**) and female’s (panel **c** and **d**) age, mediated by relative importance of *attractiveness*.

*Note:* Predicted importance of intelligence (represented by color) were obtained from OLS regressions with the interaction term of importance of attractiveness (y-axis) and age and age square (x-axis). Control variables were omitted in panel **a** and **c** and included in panel **b** and **d**.


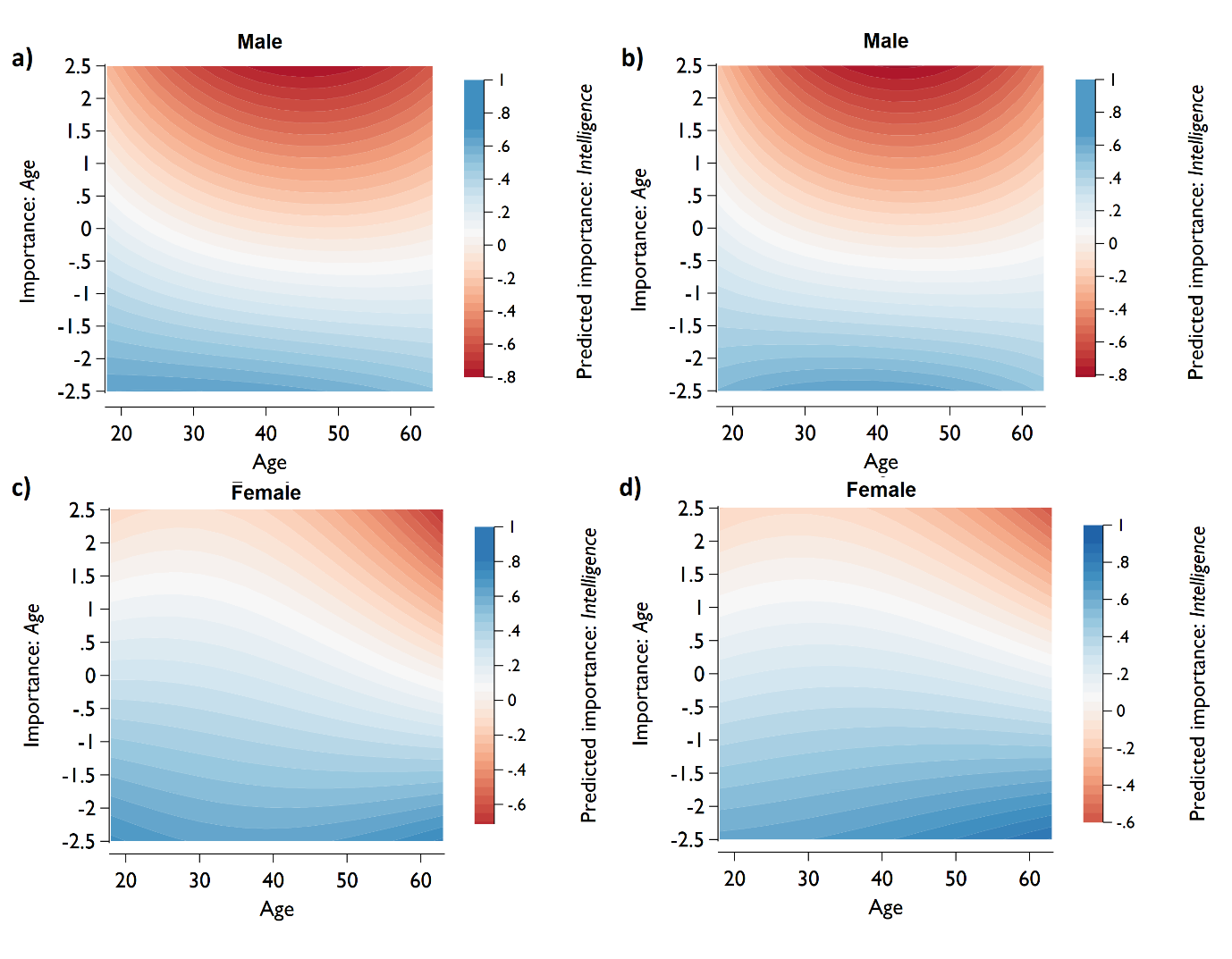


**A6 Fig.** Predicted importance of *intelligence* for sexual attraction across male’s (panel **a** and **b**) and female’s (panel **c** and **d**) age, mediated by relative importance of *age*.

*Note:* Predicted importance of intelligence (represented by color) were obtained from OLS regressions with the interaction term of importance of age (y-axis) and age and age square (x-axis). Control variables were omitted in panel **a** and **c** and included in panel **b** and **d**.


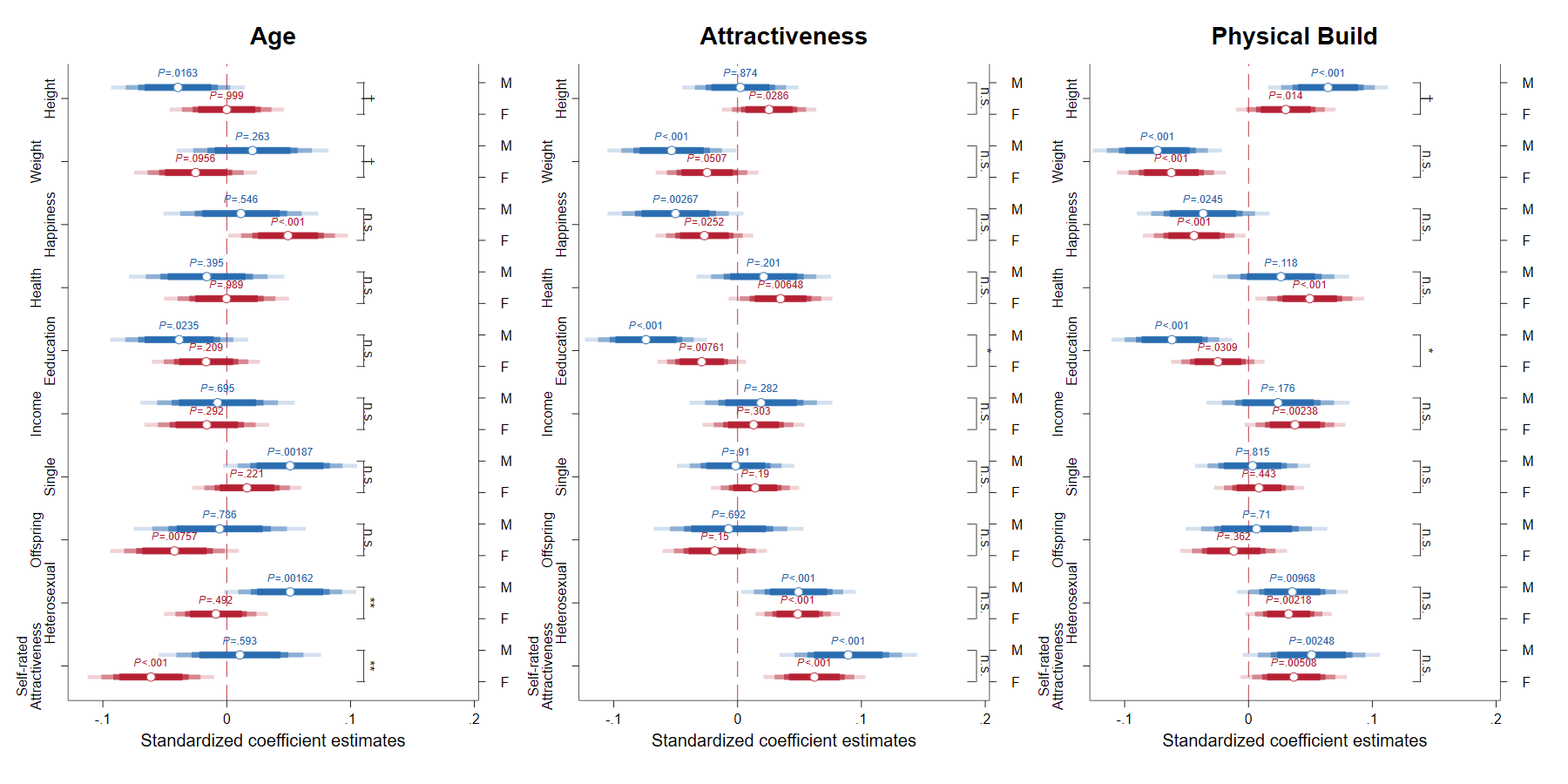


**A7 Fig.** Sex-specific factors on sexual attraction importance of aesthetic characteristics.

*Notes*: We show the effect of each independent variable on the relative importance of characteristics of interest for both sexes (effects on male and female sexual preferences are indicated by blue and red markers, respectively). P-values of the sex-specific effects are shown above the corresponding markers, with error bars indicating 90%, 95%, 99%, and 99.5% confidence intervals. The statistical significance of the sex difference for each independent variable (i.e., interaction terms with sex) is shown to the right of the coefficient estimates. † *p* < .10; * *p* < .05; ** *p* < .01; *** *p* < .001.


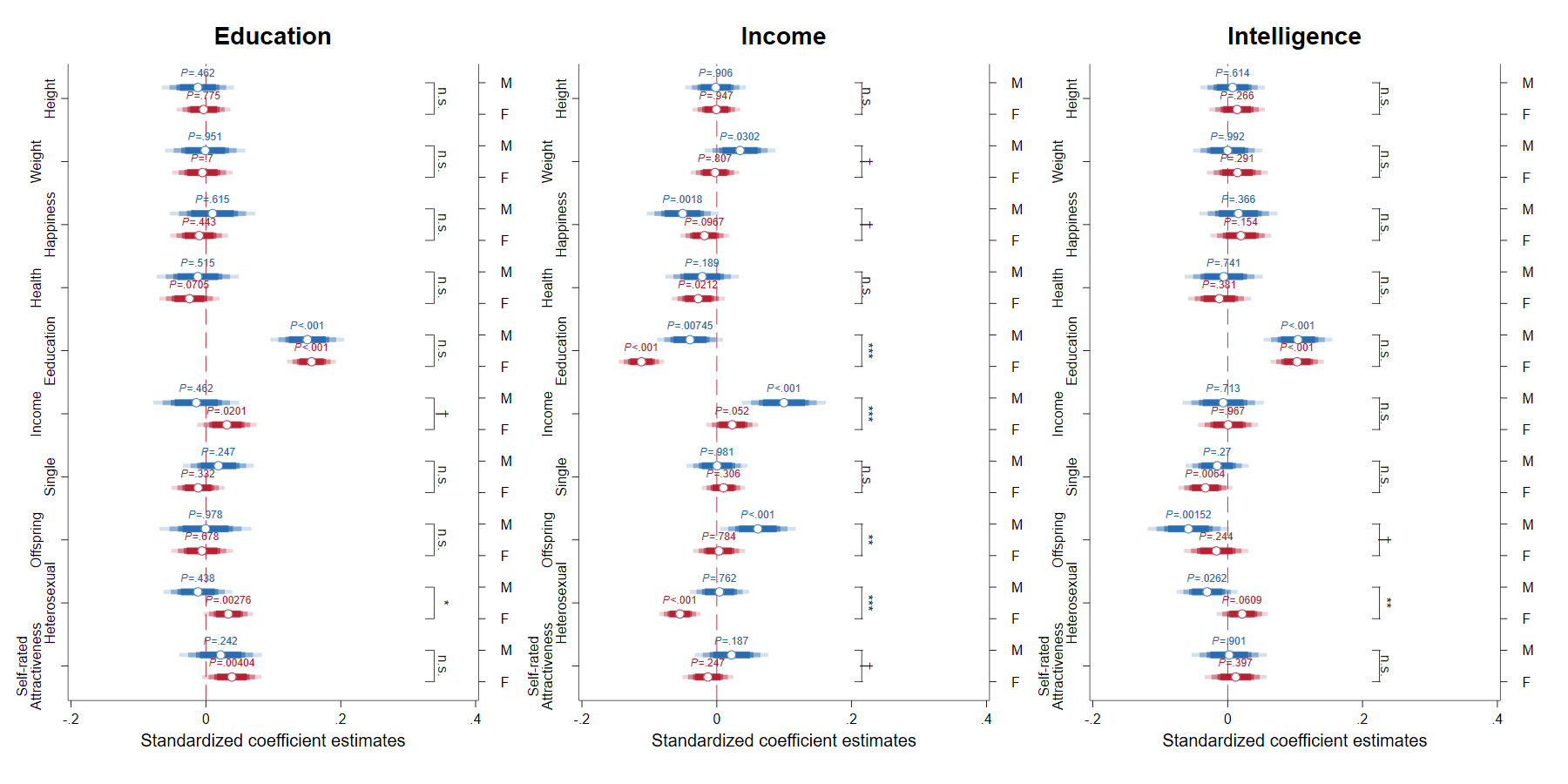


**A8 Fig.** Sex-specific factors on sexual attraction importance of resource characteristics.

*Notes*: We show the effect of each independent variable on the relative importance of characteristics of interest for both sexes (effects on male and female sexual preferences are indicated by blue and red markers, respectively). P-values of the sex-specific effects are shown above the corresponding markers, with error bars indicating 90%, 95%, 99%, and 99.5% confidence intervals. The statistical significance of the sex difference for each independent variable (i.e., interaction terms with sex) is shown to the right of the coefficient estimates. † *p* < .10; * *p* < .05; ** *p* < .01; *** *p* < .001.


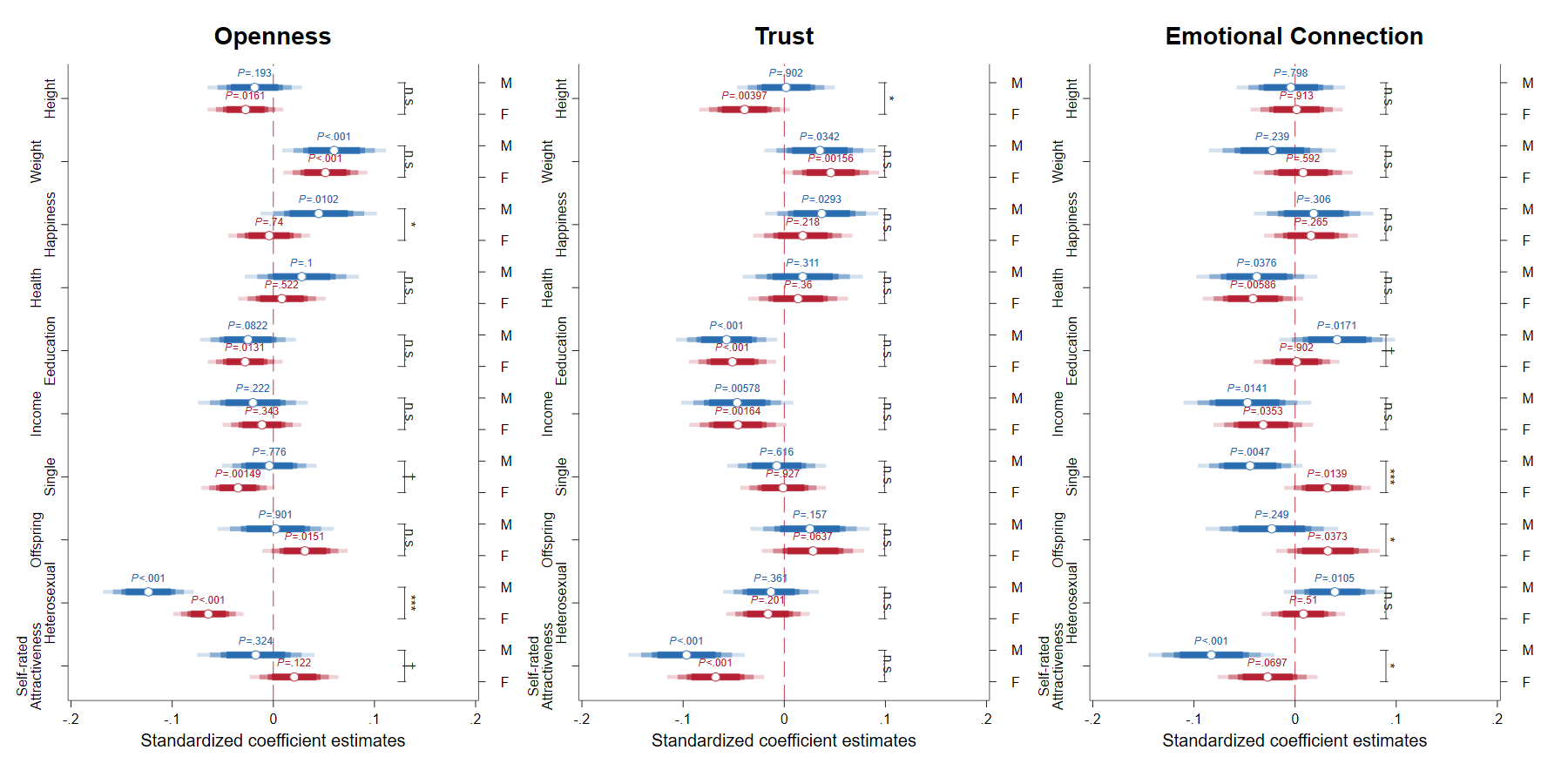


**A9 Fig.** Sex-specific factors on sexual attraction importance of personality characteristics.

*Notes*: We show the effect of each independent variable on the relative importance of characteristics of interest for both sexes (effects on male and female sexual preferences are indicated by blue and red markers, respectively). P-values of the sex-specific effects are shown above the corresponding markers, with error bars indicating 90%, 95%, 99%, and 99.5% confidence intervals. The statistical significance of the sex difference for each independent variable (i.e., interaction terms with sex) is shown to the right of the coefficient estimates. † *p* < .10; * *p* < .05; ** *p* < .01; *** *p* < .001.

**A1 Table.** Summary statistics, by sex.

|  | **Males** | | | | | **Females** | | | | |
| --- | --- | --- | --- | --- | --- | --- | --- | --- | --- | --- |
| **Variables** | *Mean* | *SD* | *Min* | *Max* | *N* | *Mean* | *SD* | *Min* | *Max* | *N* |
| Age | 41.14 | 12.15 | 18 | 64 | 4375 | 33.91 | 11.60 | 18 | 64 | 2685 |
| Height | 4.45 | 0.81 | 1 | 8 | 4375 | 3.17 | 0.83 | 1 | 8 | 2685 |
| Weight | 6.32 | 1.75 | 2 | 12 | 4375 | 5.10 | 2.10 | 1 | 12 | 2685 |
| Education |  |  |  |  |  |  |  |  |  |  |
| *< Grade 10* | 1.99% |  |  |  | 87 | 1.19% |  |  |  | 32 |
| *Grade 10* | 7.29% |  |  |  | 319 | 5.55% |  |  |  | 149 |
| *Grade 11* | 4.53% |  |  |  | 198 | 3.87% |  |  |  | 104 |
| *Grade 12* | 15.91% |  |  |  | 696 | 21.42% |  |  |  | 575 |
| *Technical College (e.g., Prevocational)* | 24.62% |  |  |  | 1077 | 16.69% |  |  |  | 448 |
| *Undergraduate (Diploma, Bachelors)* | 30.03% |  |  |  | 1314 | 36.39% |  |  |  | 977 |
| *Post Graduate (e.g., Masters)* | 13.99% |  |  |  | 612 | 13.22% |  |  |  | 355 |
| *Doctoral / PhD* | 1.65% |  |  |  | 72 | 1.68% |  |  |  | 45 |
| Income |  |  |  |  |  |  |  |  |  |  |
| *Below $20,000* | 14.19% |  |  |  | 584 | 28.68% |  |  |  | 723 |
| *$20,000 - $40,000* | 12.00% |  |  |  | 494 | 21.06% |  |  |  | 531 |
| *$40,000 - $60,000* | 16.74% |  |  |  | 689 | 21.22% |  |  |  | 535 |
| *$60,000 - $80,000* | 17.20% |  |  |  | 708 | 14.24% |  |  |  | 359 |
| *$80,000 - $100,000* | 14.87% |  |  |  | 612 | 7.34% |  |  |  | 185 |
| *$100,000 - $120,000* | 10.25% |  |  |  | 422 | 4.13% |  |  |  | 104 |
| *$120,000 - $140,000* | 4.93% |  |  |  | 203 | 1.43% |  |  |  | 36 |
| *$140,000 - $160,000* | 3.55% |  |  |  | 146 | 0.48% |  |  |  | 12 |
| *$160,000 - $180,000* | 1.51% |  |  |  | 62 | 0.44% |  |  |  | 11 |
| *$180,000 - $200,000* | 1.17% |  |  |  | 48 | 0.36% |  |  |  | 9 |
| *Above $200,000* | 3.60% |  |  |  | 148 | 0.63% |  |  |  | 16 |
| Marital Status: Single | 0.48 | 0.50 | 0 | 1 | 4375 | 0.52 | 0.50 | 0 | 1 | 2685 |
| Have Offspring | 0.53 | 0.50 | 0 | 1 | 4116 | 0.40 | 0.49 | 0 | 1 | 2521 |
| Heterosexuality | 0.66 | 0.47 | 0 | 1 | 4375 | 0.56 | 0.50 | 0 | 1 | 2685 |
| Self-rated Happiness | 68.83 | 22.53 | 0 | 100 | 4375 | 70.23 | 20.93 | 0 | 100 | 2685 |
| Self-rated Health | 73.09 | 17.70 | 0 | 100 | 4375 | 70.04 | 19.16 | 0 | 100 | 2685 |
| Self-rated Attractiveness | 61.83 | 18.13 | 0 | 100 | 4375 | 63.65 | 19.32 | 0 | 100 | 2685 |
| *Total number of observations* | 4375 |  |  |  |  | 2685 |  |  |  |  |

**A2 Table.** Correlation matrix between relative importance, by sex.

|  | **Males** | (1) | (2) | (3) | (4) | (5) | (6) | (7) | (8) | (9) |
| --- | --- | --- | --- | --- | --- | --- | --- | --- | --- | --- |
| (1) | Age | 1.00 |  |  |  |  |  |  |  |  |
| (2) | Attractiveness | 0.19*** | 1.00 |  |  |  |  |  |  |  |
| (3) | Physical build | 0.02 | 0.38*** | 1.00 |  |  |  |  |  |  |
| (4) | Intelligence | -0.28*** | -0.29*** | -0.23*** | 1.00 |  |  |  |  |  |
| (5) | Income | -0.18*** | -0.05** | -0.02 | -0.22*** | 1.00 |  |  |  |  |
| (6) | Education | -0.21*** | -0.19*** | -0.14*** | 0.24*** | -0.18*** | 1.00 |  |  |  |
| (7) | Trust | -0.24*** | -0.40*** | -0.36*** | -0.08*** | -0.03* | -0.22*** | 1.00 |  |  |
| (8) | Openness | -0.18*** | -0.16*** | -0.21*** | -0.13*** | -0.03† | -0.13*** | -0.00 | 1.00 |  |
| (9) | Emotional connection | -0.22*** | -0.33*** | -0.29*** | -0.07*** | -0.12*** | -0.19*** | 0.18*** | -0.11*** | 1.00 |
|  | **Females** | (1) | (2) | (3) | (4) | (5) | (6) | (7) | (8) | (9) |
| (1) | Age | 1.00 |  |  |  |  |  |  |  |  |
| (2) | Attractiveness | 0.10*** | 1.00 |  |  |  |  |  |  |  |
| (3) | Physical build | -0.05** | 0.38*** | 1.00 |  |  |  |  |  |  |
| (4) | Intelligence | -0.19*** | -0.28*** | -0.21*** | 1.00 |  |  |  |  |  |
| (5) | Income | -0.20*** | -0.13*** | -0.08*** | -0.09*** | 1.00 |  |  |  |  |
| (6) | Education | -0.15*** | -0.25*** | -0.23*** | 0.23*** | -0.14*** | 1.00 |  |  |  |
| (7) | Trust | -0.24*** | -0.33*** | -0.30*** | -0.15*** | -0.03 | -0.21*** | 1.00 |  |  |
| (8) | Openness | -0.20*** | -0.14*** | -0.15*** | -0.15*** | -0.13*** | -0.16*** | 0.05** | 1.00 |  |
| (9) | Emotional connection | -0.19*** | -0.31*** | -0.28*** | -0.13*** | -0.10*** | -0.17*** | 0.21*** | -0.07*** | 1.00 |

Notes: *N_males_*= 4,375; *N_females_* = 2,685. †p < .10; *p < .05; **p < .01; ***p < .001.

**A3 Table.** Principal component analysis with varimax rotation factor loadings of importance of characteristics of sexual attraction.

|  | Factors | | |  |
| --- | --- | --- | --- | --- |
| **Characteristics** | Aesthetic | Resource | Personality | Uniqueness |
| Age | **0.58** | 0.37 | 0.09 | 0.52 |
| Attractiveness | **0.89** | 0.11 | 0.05 | 0.19 |
| Physical build/ features | **0.85** | 0.12 | 0.03 | 0.26 |
| Intelligence | 0.16 | **0.82** | 0.16 | 0.28 |
| Income | 0.10 | **0.67** | 0.41 | 0.37 |
| Education | 0.18 | **0.76** | -0.04 | 0.39 |
| Trust | 0.23 | -0.03 | **0.68** | 0.48 |
| Openness | -0.04 | 0.14 | **0.80** | 0.33 |
| Emotional connection | 0.04 | 0.24 | **0.73** | 0.41 |

Notes: Factor loadings (absolute value) higher than .5 are in bold. *n* = 7,060. Three principal-component factors are retained with eigenvalue above 1. The eigenvalue of the three factors equals to 3.13, 1.56, and 1.07 for *aesthetic, resource,* and *personality*, respectively.

**A4 Table.** Sex differences of mating preference (relative importance of *aesthetic*, *resource*, and *personality* characteristics to sexual attraction) across life span – cubic age effect.

|  | **Aesthetic** | Age | Attractiveness | Physical  Build | **Resource** | Education | Intelligence | Income | **Personality** | Openness | Trust | Emotional  Connection |
| --- | --- | --- | --- | --- | --- | --- | --- | --- | --- | --- | --- | --- |
|  | (1) | (2) | (3) | (4) | (5) | (6) | (7) | (8) | (9) | (10) | (11) | (12) |
| Male | .17 | -.61 | .034 | 1.2* | 1.1 | -.8 | .2 | .15 | -1.2† | -.11 | -.31 | .28 |
|  | (0.279) | (-0.946) | (0.063) | (2.129) | (1.600) | (-1.305) | (0.362) | (0.277) | (-1.904) | (-0.196) | (-0.509) | (0.433) |
| Age | .033 | -.13** | .038 | .096** | 2.1e-03 | -.15*** | -.037 | .079* | -.036 | .055 | 2.3e-05 | .049 |
|  | (0.811) | (-2.937) | (1.003) | (2.613) | (0.048) | (-3.593) | (-0.976) | (2.017) | (-0.822) | (1.475) | (0.001) | (1.110) |
| Age^2^ | -6.7e-04 | 3.0e-03** | -1.1e-03 | -2.1e-03* | -7.4e-04 | 3.0e-03* | 1.1e-03 | -1.9e-03† | 1.4e-03 | -1.1e-03 | 1.8e-04 | -1.1e-03 |
|  | (-0.597) | (2.585) | (-1.066) | (-2.054) | (-0.609) | (2.518) | (1.070) | (-1.791) | (1.192) | (-1.030) | (0.156) | (-0.929) |
| Age^3^ | 4.1e-06 | -2.4e-05* | 9.3e-06 | 1.4e-05† | 1.0e-05 | -1.8e-05† | -1.1e-05 | 1.5e-05 | -1.4e-05 | 6.7e-06 | -2.0e-06 | 8.4e-06 |
|  | (0.422) | (-2.350) | (1.036) | (1.658) | (0.974) | (-1.722) | (-1.179) | (1.617) | (-1.394) | (0.761) | (-0.204) | (0.784) |
| Male*Age | .021 | .044 | .019 | -.06 | -.11† | .06 | -.018 | -.016 | .088 | 6.0e-03 | 5.7e-03 | -.041 |
|  | (0.407) | (0.796) | (0.413) | (-1.301) | (-1.950) | (1.146) | (-0.374) | (-0.340) | (1.600) | (0.128) | (0.109) | (-0.735) |
| Male*Age^2^ | -6.3e-04 | -1.2e-03 | -3.1e-04 | 1.4e-03 | 2.9e-03† | -1.5e-03 | 1.4e-05 | 5.5e-04 | -2.2e-03 | -1.9e-05 | 1.9e-05 | 9.9e-04 |
|  | (-0.444) | (-0.791) | (-0.249) | (1.113) | (1.915) | (-1.027) | (0.010) | (0.431) | (-1.526) | (-0.015) | (0.013) | (0.663) |
| Male*Age^3^ | 4.8e-06 | 9.7e-06 | 6.9e-07 | -1.0e-05 | -2.3e-05† | 1.1e-05 | 3.4e-06 | -5.6e-06 | 1.9e-05 | -1.9e-07 | -8.0e-07 | -7.3e-06 |
|  | (0.401) | (0.778) | (0.064) | (-1.000) | (-1.848) | (0.877) | (0.306) | (-0.510) | (1.494) | (-0.018) | (-0.066) | (-0.568) |
| Height | .021* | -.019† | .017† | .043*** | -.011 | -5.7e-03 | .013 | -3.9e-03 | -.01 | -.022* | -.023* | 4.1e-05 |
|  | (2.036) | (-1.730) | (1.837) | (4.627) | (-0.986) | (-0.588) | (1.396) | (-0.461) | (-0.963) | (-2.502) | (-2.248) | (0.004) |
| Weight | -.063*** | -5.4e-03 | -.035*** | -.068*** | .023† | -1.5e-03 | 1.0e-02 | 8.3e-03 | .04*** | .055*** | .04*** | -4.8e-03 |
|  | (-5.487) | (-0.460) | (-3.530) | (-6.647) | (1.905) | (-0.134) | (0.965) | (0.906) | (3.404) | (5.630) | (3.666) | (-0.406) |
| Happiness | -.036** | .036** | -.036*** | -.042*** | 1.0e-04 | -3.1e-03 | .017 | -.028** | .036** | .013 | .026* | .017 |
|  | (-3.174) | (3.120) | (-3.691) | (-4.185) | (0.009) | (-0.289) | (1.558) | (-3.001) | (3.088) | (1.294) | (2.253) | (1.493) |
| Health | .046*** | -5.5e-03 | .029** | .04*** | -.064*** | -.02† | -.011 | -.025* | .018 | .015 | .016 | -.039*** |
|  | (3.930) | (-0.458) | (2.909) | (3.865) | (-5.392) | (-1.794) | (-1.030) | (-2.545) | (1.524) | (1.433) | (1.387) | (-3.336) |
| Education | -.055*** | -.024* | -.045*** | -.039*** | .069*** | .15*** | .1*** | -.086*** | -.014 | -.026** | -.053*** | .016 |
|  | (-5.478) | (-2.288) | (-5.131) | (-4.249) | (6.419) | (16.794) | (10.873) | (-10.117) | (-1.302) | (-2.976) | (-5.289) | (1.519) |
| Income | .025* | -.014 | .015 | .031** | .042*** | .015 | 1.5e-03 | .048*** | -.068*** | -.013 | -.047*** | -.037** |
|  | (2.247) | (-1.131) | (1.520) | (3.111) | (3.623) | (1.326) | (0.141) | (4.573) | (-5.805) | (-1.319) | (-4.260) | (-3.175) |
| Marital Status: Single | .016 | .032** | 7.4e-03 | 6.7e-03 | .022* | -1.1e-03 | -.028** | .01 | -.038*** | -.024** | -3.7e-03 | 4.6e-04 |
|  | (1.628) | (3.054) | (0.848) | (0.767) | (2.059) | (-0.114) | (-3.003) | (1.269) | (-3.688) | (-2.733) | (-0.385) | (0.046) |
| Have Offspring | -.012 | -.03* | -.014 | -4.3e-03 | -.013 | 1.6e-03 | -.028* | .014 | .025* | .024* | .027* | .01 |
|  | (-0.958) | (-2.397) | (-1.314) | (-0.421) | (-1.048) | (0.141) | (-2.519) | (1.421) | (1.990) | (2.352) | (2.284) | (0.864) |
| Heterosexual | .034*** | .012 | .048*** | .032*** | .033** | .017† | 3.1e-03 | -.034*** | -.067*** | -.085*** | -.015 | .022* |
|  | (3.560) | (1.224) | (5.773) | (3.876) | (3.273) | (1.836) | (0.350) | (-4.411) | (-6.736) | (-10.268) | (-1.604) | (2.280) |
| Self-rated Attractiveness | .06*** | -.036** | .072*** | .042*** | 3.7e-03 | .033** | .011 | -2.6e-03 | -.064*** | 8.7e-03 | -.079*** | -.049*** |
|  | (5.190) | (-2.943) | (7.174) | (4.091) | (0.304) | (3.061) | (1.007) | (-0.270) | (-5.317) | (0.821) | (-7.075) | (-4.158) |
| Constant | -.71 | 1.5** | -.26 | -1.3** | .51 | 2*** | .67 | -2.5*** | .2 | -.5 | .47 | -.078 |
|  | (-1.502) | (3.082) | (-0.596) | (-3.156) | (1.003) | (4.043) | (1.531) | (-5.525) | (0.400) | (-1.154) | (1.005) | (-0.155) |
| Observations | 6637 | 6625 | 6625 | 6625 | 6637 | 6625 | 6625 | 6625 | 6637 | 6625 | 6625 | 6625 |
| *R*^2^ | 0.070 | 0.029 | 0.078 | 0.087 | 0.052 | 0.087 | 0.053 | 0.038 | 0.041 | 0.070 | 0.040 | 0.026 |
| F | 30.82 | 12.27 | 34.17 | 37.32 | 22.79 | 40.55 | 22.40 | 14.60 | 16.70 | 28.16 | 16.18 | 10.90 |

Notes: OLS regressions. *t*-statistics in parentheses (standard errors adjusted for heteroscedasticity). Standardized regression coefficients in italics. † *p* < .10; * *p* < .05; ** *p* < .01; *** *p* < .001.

**A5 Table.** Sex differences of mating preference (relative importance of *aesthetic*, *resource*, and *personality* characteristics to sexual attraction) across life span – quartic age effect.

|  | **Aesthetic** | Age | Attractiveness | Physical  Build | **Resource** | Education | Intelligence | Income | **Personality** | Openness | Trust | Emotional  Connection |
| --- | --- | --- | --- | --- | --- | --- | --- | --- | --- | --- | --- | --- |
|  | (1) | (2) | (3) | (4) | (5) | (6) | (7) | (8) | (9) | (10) | (11) | (12) |
| Male | .47* | -.3 | .16 | .72*** | -.14 | -.39* | .29 | -8.3e-03 | -.33 | -.055 | -.38† | -.041 |
|  | (2.467) | (-1.472) | (0.921) | (4.137) | (-0.661) | (-2.032) | (1.597) | (-0.048) | (-1.622) | (-0.311) | (-1.943) | (-0.205) |
| Age | .016† | -.028** | -6.6e-04 | .037*** | -.039*** | -.081*** | 6.9e-03 | .016† | .023* | .028*** | 8.3e-03 | .014 |
|  | (1.875) | (-2.988) | (-0.081) | (4.619) | (-4.083) | (-9.115) | (0.826) | (1.889) | (2.417) | (3.416) | (0.954) | (1.516) |
| Age^2^ | -2.0e-04† | 3.0e-04* | -3.2e-05 | -4.1e-04*** | 4.3e-04*** | 9.4e-04*** | -1.1e-04 | -1.8e-04 | -2.3e-04† | -2.8e-04** | -5.6e-05 | -1.7e-04 |
|  | (-1.730) | (2.407) | (-0.299) | (-3.912) | (3.319) | (7.969) | (-1.016) | (-1.588) | (-1.803) | (-2.649) | (-0.482) | (-1.366) |
| Male*Age | -3.1e-03 | .012 | .011 | -.019* | -5.6e-03 | .021† | -.028** | 1.6e-03 | 8.7e-03 | 3.3e-03 | .011 | -.011 |
|  | (-0.289) | (1.007) | (1.144) | (-1.997) | (-0.478) | (1.951) | (-2.776) | (0.164) | (0.758) | (0.333) | (0.975) | (-1.010) |
| Male*Age^2^ | -1.3e-05 | -1.5e-04 | -1.6e-04 | 2.0e-04 | 8.4e-05 | -2.9e-04* | 3.5e-04** | -2.2e-05 | -7.1e-05 | 4.9e-06 | -9.2e-05 | 1.6e-04 |
|  | (-0.094) | (-1.009) | (-1.292) | (1.604) | (0.548) | (-2.102) | (2.662) | (-0.172) | (-0.473) | (0.039) | (-0.656) | (1.117) |
| Height | .021* | -.018† | .016† | .043*** | -.01 | -5.4e-03 | .013 | -4.3e-03 | -.01 | -.022* | -.023* | -2.8e-05 |
|  | (2.008) | (-1.680) | (1.800) | (4.604) | (-0.952) | (-0.558) | (1.424) | (-0.503) | (-0.970) | (-2.528) | (-2.240) | (-0.003) |
| Weight | -.062*** | -7.5e-03 | -.034*** | -.067*** | .022† | -2.7e-03 | 9.0e-03 | 9.7e-03 | .04*** | .056*** | .04*** | -4.3e-03 |
|  | (-5.429) | (-0.635) | (-3.422) | (-6.549) | (1.877) | (-0.252) | (0.871) | (1.053) | (3.376) | (5.707) | (3.646) | (-0.367) |
| Happiness | -.037** | .037** | -.036*** | -.042*** | 6.6e-04 | -2.9e-03 | .017 | -.028** | .036** | .013 | .026* | .017 |
|  | (-3.207) | (3.166) | (-3.734) | (-4.190) | (0.056) | (-0.266) | (1.584) | (-3.033) | (3.072) | (1.269) | (2.263) | (1.492) |
| Health | .046*** | -4.5e-03 | .029** | .04*** | -.064*** | -.019† | -.011 | -.026** | .018 | .015 | .016 | -.039*** |
|  | (3.891) | (-0.380) | (2.855) | (3.833) | (-5.352) | (-1.746) | (-0.989) | (-2.608) | (1.524) | (1.399) | (1.402) | (-3.351) |
| Education | -.054*** | -.027** | -.044*** | -.037*** | .069*** | .15*** | .1*** | -.084*** | -.015 | -.025** | -.054*** | .017 |
|  | (-5.395) | (-2.602) | (-4.985) | (-4.118) | (6.414) | (16.637) | (10.795) | (-9.872) | (-1.379) | (-2.854) | (-5.350) | (1.601) |
| Income | .026* | -.014 | .016 | .032** | .042*** | .014 | 1.1e-03 | .048*** | -.068*** | -.013 | -.047*** | -.037** |
|  | (2.271) | (-1.201) | (1.560) | (3.146) | (3.617) | (1.280) | (0.106) | (4.621) | (-5.822) | (-1.291) | (-4.275) | (-3.157) |
| Marital Status: Single | .015 | .034*** | 6.0e-03 | 5.4e-03 | .022* | 6.1e-04 | -.027** | 8.4e-03 | -.038*** | -.025** | -3.4e-03 | -2.1e-04 |
|  | (1.547) | (3.315) | (0.696) | (0.624) | (2.088) | (0.064) | (-2.877) | (1.054) | (-3.635) | (-2.850) | (-0.353) | (-0.021) |
| Have Offspring | -.012 | -.028* | -.015 | -5.3e-03 | -.013 | 2.8e-03 | -.028* | .012 | .025* | .024* | .027* | 1.0e-02 |
|  | (-1.010) | (-2.250) | (-1.405) | (-0.510) | (-1.043) | (0.248) | (-2.441) | (1.294) | (2.036) | (2.291) | (2.309) | (0.825) |
| Heterosexual | .034*** | .013 | .048*** | .032*** | .033** | .017† | 3.4e-03 | -.034*** | -.067*** | -.086*** | -.015 | .022* |
|  | (3.556) | (1.286) | (5.755) | (3.832) | (3.237) | (1.890) | (0.381) | (-4.458) | (-6.693) | (-10.298) | (-1.600) | (2.253) |
| Self-rated Attractiveness | .06*** | -.036** | .072*** | .042*** | 2.9e-03 | .033** | .011 | -2.3e-03 | -.063*** | 8.9e-03 | -.079*** | -.049*** |
|  | (5.227) | (-2.974) | (7.215) | (4.087) | (0.241) | (3.051) | (0.986) | (-0.240) | (-5.286) | (0.843) | (-7.089) | (-4.167) |
| Constant | -.52*** | .43** | .17 | -.68*** | .97*** | 1.2*** | .18 | -1.8*** | -.45** | -.19 | .37* | .31† |
|  | (-3.399) | (2.625) | (1.198) | (-4.833) | (5.779) | (7.509) | (1.244) | (-11.964) | (-2.699) | (-1.338) | (2.459) | (1.860) |
| Observations | 6637 | 6625 | 6625 | 6625 | 6637 | 6625 | 6625 | 6625 | 6637 | 6625 | 6625 | 6625 |
| *R*^2^ | 0.070 | 0.028 | 0.078 | 0.087 | 0.051 | 0.087 | 0.053 | 0.037 | 0.041 | 0.070 | 0.040 | 0.026 |
| F | 34.85 | 13.22 | 38.34 | 42.24 | 25.41 | 45.60 | 25.28 | 16.29 | 18.76 | 31.82 | 18.33 | 12.29 |

Notes: OLS regressions. *t*-statistics in parentheses (standard errors adjusted for heteroscedasticity). Standardized regression coefficients in italics. † *p* < .10; * *p* < .05; ** *p* < .01; *** *p* < .001.

**A6 Table.** Sex differences of mating preference (relative importance of *aesthetic*, *resource*, and *personality* characteristics to sexual attraction) across life span – linear age effect.

|  | **Aesthetic** | Age | Attractiveness | Physical  Build | **Resource** | Education | Intelligence | Income | **Personality** | Openness | Trust | Emotional  Connection |
| --- | --- | --- | --- | --- | --- | --- | --- | --- | --- | --- | --- | --- |
|  | (1) | (2) | (3) | (4) | (5) | (6) | (7) | (8) | (9) | (10) | (11) | (12) |
| Male | .55*** | -.16* | .41*** | .54*** | -.38*** | -.22*** | -.2*** | .073 | -.17* | .015 | -.22*** | -.23*** |
|  | (8.755) | (-2.420) | (7.311) | (9.502) | (-5.598) | (-3.446) | (-3.392) | (1.334) | (-2.503) | (0.253) | (-3.571) | (-3.641) |
| Age | 1.1e-03 | -5.5e-03*** | -3.5e-03* | 5.9e-03*** | -6.7e-03*** | -1.0e-02*** | -9.4e-04 | 2.2e-03† | 5.5e-03*** | 6.2e-03*** | 3.9e-03** | 1.7e-03 |
|  | (0.759) | (-3.471) | (-2.503) | (4.450) | (-4.053) | (-6.482) | (-0.671) | (1.685) | (3.509) | (4.662) | (2.647) | (1.104) |
| Male*Age | -5.5e-03*** | 1.5e-03 | -2.3e-03 | -5.9e-03*** | 4.0e-03* | 3.8e-03* | -6.4e-04 | -1.4e-03 | 1.5e-03 | 1.7e-03 | 2.7e-03† | 5.8e-04 |
|  | (-3.357) | (0.843) | (-1.543) | (-4.041) | (2.222) | (2.270) | (-0.424) | (-0.966) | (0.853) | (1.182) | (1.666) | (0.350) |
| Height | .019† | -.017 | .016† | .041*** | -7.4e-03 | -3.7e-04 | .014 | -5.5e-03 | -.012 | -.024** | -.023* | -6.4e-04 |
|  | (1.887) | (-1.547) | (1.729) | (4.390) | (-0.681) | (-0.038) | (1.453) | (-0.641) | (-1.120) | (-2.718) | (-2.304) | (-0.061) |
| Weight | -.058*** | -.012 | -.031** | -.06*** | .012 | -.019† | 6.8e-03 | .014 | .046*** | .062*** | .042*** | -2.8e-03 |
|  | (-5.093) | (-1.020) | (-3.168) | (-5.984) | (1.021) | (-1.758) | (0.668) | (1.520) | (3.896) | (6.355) | (3.910) | (-0.238) |
| Happiness | -.04*** | .039*** | -.039*** | -.045*** | 7.7e-03 | 6.9e-03 | .02† | -.031*** | .032** | 8.9e-03 | .024* | .016 |
|  | (-3.480) | (3.385) | (-3.998) | (-4.562) | (0.645) | (0.634) | (1.837) | (-3.349) | (2.738) | (0.885) | (2.105) | (1.462) |
| Health | .045*** | -4.7e-03 | .028** | .04*** | -.063*** | -.019† | -.01 | -.026** | .018 | .014 | .016 | -.039*** |
|  | (3.872) | (-0.392) | (2.819) | (3.840) | (-5.292) | (-1.725) | (-0.926) | (-2.626) | (1.501) | (1.381) | (1.383) | (-3.327) |
| Education | -.053*** | -.029** | -.043*** | -.035*** | .066*** | .15*** | .1*** | -.083*** | -.013 | -.023** | -.053*** | .018† |
|  | (-5.275) | (-2.788) | (-4.960) | (-3.830) | (6.148) | (15.833) | (10.857) | (-9.754) | (-1.228) | (-2.638) | (-5.316) | (1.706) |
| Income | .034** | -.021† | .022* | .042*** | .024* | -.013 | -4.3e-03 | .055*** | -.057*** | -2.3e-03 | -.043*** | -.035** |
|  | (3.037) | (-1.842) | (2.188) | (4.247) | (2.099) | (-1.189) | (-0.409) | (5.576) | (-5.087) | (-0.244) | (-3.966) | (-3.131) |
| Marital Status: Single | .013 | .036*** | 4.5e-03 | 2.8e-03 | .027* | 7.8e-03 | -.025** | 6.5e-03 | -.04*** | -.027** | -4.6e-03 | -7.2e-04 |
|  | (1.345) | (3.497) | (0.522) | (0.321) | (2.545) | (0.812) | (-2.727) | (0.814) | (-3.903) | (-3.161) | (-0.479) | (-0.072) |
| Have Offspring | -7.1e-03 | -.033** | -.011 | 1.8e-03 | -.025* | -.016 | -.03** | .017† | .032** | .03** | .03** | .012 |
|  | (-0.590) | (-2.673) | (-1.086) | (0.179) | (-2.004) | (-1.401) | (-2.732) | (1.818) | (2.606) | (2.995) | (2.584) | (0.967) |
| Heterosexual | .032*** | .015 | .047*** | .029*** | .038*** | .026** | 4.3e-03 | -.037*** | -.069*** | -.089*** | -.016† | .021* |
|  | (3.328) | (1.531) | (5.616) | (3.443) | (3.758) | (2.842) | (0.486) | (-4.748) | (-7.018) | (-10.678) | (-1.734) | (2.180) |
| Self-rated Attractiveness | .061*** | -.037** | .073*** | .042*** | 1.8e-03 | .032** | 9.6e-03 | -1.9e-03 | -.063*** | 9.5e-03 | -.079*** | -.049*** |
|  | (5.260) | (-2.977) | (7.275) | (4.089) | (0.149) | (2.943) | (0.895) | (-0.198) | (-5.227) | (0.890) | (-7.059) | (-4.190) |
| Constant | -.26*** | .05 | .22*** | -.16*** | .41*** | -.041 | .3*** | -1.5*** | -.15** | .17*** | .45*** | .52*** |
|  | (-4.962) | (0.885) | (4.614) | (-3.300) | (7.102) | (-0.765) | (6.111) | (-32.034) | (-2.647) | (3.653) | (8.822) | (9.640) |
| Observations | 6637 | 6625 | 6625 | 6625 | 6637 | 6625 | 6625 | 6625 | 6637 | 6625 | 6625 | 6625 |
| *R*^2^ | 0.069 | 0.027 | 0.077 | 0.084 | 0.046 | 0.070 | 0.051 | 0.036 | 0.039 | 0.067 | 0.040 | 0.026 |
| F | 39.53 | 14.67 | 43.56 | 46.84 | 25.93 | 41.80 | 28.33 | 17.89 | 20.61 | 36.07 | 20.95 | 14.07 |

Notes: OLS regressions. *t*-statistics in parentheses (standard errors adjusted for heteroscedasticity). Standardized regression coefficients in italics. † *p* < .10; * *p* < .05; ** *p* < .01; *** *p* < .001.

**A7 Table.** Sex-specific effects on the importance of aesthetics on sexual attraction.

|  | *Males* | *Females* | *Males* | *Females* | *Males* | *Females* | *Males* | *Females* |
| --- | --- | --- | --- | --- | --- | --- | --- | --- |
|  | (1) | (2) | (3) | (4) | (5) | (6) | (7) | (8) |
| *Dep. Var.* | Aesthetic | | Age | | Attractiveness | | Physical Build | |
| Age | .014† | .017† | -.015† | -.03** | .01† | 2.6e-03 | .016* | .042*** |
|  | (1.910) | (1.807) | (-1.885) | (-3.005) | (1.654) | (0.285) | (2.505) | (4.723) |
|  | *.215* | *.263* | *-.219* | *-.431* | *.193* | *.041* | *.291* | *.687* |
| Age*Age | -2.2e-04* | -2.2e-04† | 1.4e-04 | 3.0e-04* | -1.9e-04* | -7.6e-05 | -1.9e-04* | -4.7e-04*** |
|  | (-2.460) | (-1.816) | (1.473) | (2.297) | (-2.575) | (-0.661) | (-2.547) | (-4.186) |
|  | *-.27* | *-.25* | *.165* | *.314* | *-.293* | *-.091* | *-.286* | *-.574* |
| Height | .029† | .022 | 1.6e-03 | -.047* | .03* | 2.8e-03 | .035* | .078*** |
|  | (1.709) | (1.166) | (0.092) | (-2.412) | (2.124) | (0.163) | (2.330) | (4.412) |
|  | *.029* | *.024* | *1.6e-03* | *-.048* | *.037* | *3.3e-03* | *.043* | *.092* |
| Weight | -.033*** | -.034*** | -.016† | 9.5e-03 | -.013† | -.025*** | -.034*** | -.036*** |
|  | (-3.766) | (-4.123) | (-1.821) | (1.072) | (-1.838) | (-3.308) | (-4.448) | (-4.786) |
|  | *-.072* | *-.095* | *-.034* | *.025* | *-.035* | *-.074* | *-.088* | *-.109* |
| Happiness | -1.3e-03* | -2.3e-03** | 2.3e-03*** | 5.3e-04 | -1.2e-03* | -2.4e-03** | -2.0e-03*** | -1.7e-03* |
|  | (-1.962) | (-2.741) | (3.464) | (0.580) | (-2.315) | (-2.986) | (-3.619) | (-2.183) |
|  | *-.036* | *-.064* | *.063* | *.013* | *-.042* | *-.07* | *-.068* | *-.051* |
| Health | 3.2e-03*** | 1.4e-03 | 6.3e-05 | -8.2e-04 | 1.9e-03** | 1.1e-03 | 2.7e-03*** | 1.3e-03 |
|  | (3.661) | (1.481) | (0.073) | (-0.820) | (2.659) | (1.255) | (3.642) | (1.526) |
|  | *.07* | *.035* | *1.4e-03* | *-.019* | *.051* | *.029* | *.072* | *.036* |
| Education | -.025** | -.056*** | -.013 | -.028* | -.018* | -.05*** | -.015* | -.044*** |
|  | (-2.951) | (-5.103) | (-1.449) | (-2.413) | (-2.534) | (-4.818) | (-2.022) | (-4.251) |
|  | *-.047* | *-.107* | *-.024* | *-.05* | *-.042* | *-.101* | *-.034* | *-.089* |
| Income | .011† | .016 | -7.2e-03 | -3.8e-03 | 5.6e-03 | .01 | .016** | .011 |
|  | (1.709) | (1.639) | (-1.154) | (-0.354) | (1.099) | (1.062) | (3.262) | (1.135) |
|  | *.032* | *.038* | *-.022* | *-8.3e-03* | *.021* | *.026* | *.06* | *.029* |
| Marital Status: Single | .036 | .022 | .037 | .11** | .026 | -6.7e-03 | .014 | 7.1e-03 |
|  | (1.367) | (0.716) | (1.373) | (3.256) | (1.195) | (-0.232) | (0.627) | (0.251) |
|  | *.023* | *.015* | *.023* | *.066* | *.02* | *-4.7e-03* | *.01* | *5.1e-03* |
| Have Offspring | -.047 | .013 | -.083** | -4.9e-03 | -.038 | -.019 | -.022 | 2.6e-03 |
|  | (-1.503) | (0.312) | (-2.618) | (-0.115) | (-1.485) | (-0.502) | (-0.834) | (0.074) |
|  | *-.029* | *8.2e-03* | *-.051* | *-3.0e-03* | *-.029* | *-.013* | *-.016* | *1.8e-03* |
| Heterosexual | .069** | .08** | -.018 | .1** | .1*** | .098*** | .067** | .073** |
|  | (2.614) | (2.676) | (-0.673) | (3.194) | (4.672) | (3.467) | (3.006) | (2.639) |
|  | *.041* | *.053* | *-.011* | *.064* | *.074* | *.069* | *.047* | *.052* |
| Self-rated Attractiveness | 3.0e-03*** | 3.8e-03*** | -3.5e-03*** | 5.8e-04 | 3.5e-03*** | 4.6e-03*** | 2.1e-03** | 2.6e-03** |
|  | (3.544) | (4.099) | (-4.033) | (0.565) | (5.041) | (5.265) | (2.896) | (3.011) |
|  | *.066* | *.097* | *-.077* | *.014* | *.095* | *.125* | *.056* | *.072* |
| Constant | -.28 | -.4* | .35† | .64** | .013 | .22 | -.09 | -.82*** |
|  | (-1.526) | (-2.177) | (1.836) | (3.260) | (0.082) | (1.300) | (-0.568) | (-4.805) |
| Observations | 4116 | 2521 | 4106 | 2519 | 4106 | 2519 | 4106 | 2519 |
| *R*^2^ | 0.031 | 0.034 | 0.021 | 0.032 | 0.040 | 0.038 | 0.028 | 0.049 |
| F | 10.94 | 7.570 | 6.979 | 7.217 | 14.10 | 8.342 | 9.425 | 10.54 |

*Notes*: OLS regressions. *t*-statistics in parentheses (standard errors adjusted for heteroscedasticity). Standardized regression coefficients in italics. † *p* < .10; * *p* < .05; ** *p* < .01; *** *p* < .001.

**A8 Table.** Sex-specific effects on the importance of resources on sexual attraction.

|  | *Males* | *Females* | *Males* | *Females* | *Males* | *Females* | *Males* | *Females* |
| --- | --- | --- | --- | --- | --- | --- | --- | --- |
|  | (1) | (2) | (3) | (4) | (5) | (6) | (5) | (6) |
| *Dep. Var.* | Resource | | Intelligence | | Income | | Education | |
| Age | -.044*** | -.044*** | -.023*** | .013 | .025*** | -4.9e-03 | -.063*** | -.077*** |
|  | (-5.584) | (-4.221) | (-3.329) | (1.443) | (4.163) | (-0.535) | (-9.007) | (-7.729) |
|  | *-.649* | *-.614* | *-.38* | *.217* | *.499* | *-.084* | *-1.03* | *-1.11* |
| Age*Age | 5.1e-04*** | 4.6e-04*** | 2.6e-04** | -1.7e-04 | -2.8e-04*** | 3.3e-05 | 6.8e-04*** | 9.0e-04*** |
|  | (5.415) | (3.383) | (3.113) | (-1.448) | (-3.965) | (0.283) | (8.274) | (7.028) |
|  | *.61* | *.472* | *.344* | *-.206* | *-.453* | *.042* | *.908* | *.955* |
| Height | 6.9e-03 | -.035† | .017 | 9.1e-03 | -7.8e-04 | -2.5e-03 | -3.1e-03 | -.015 |
|  | (0.402) | (-1.740) | (1.117) | (0.521) | (-0.059) | (-0.153) | (-0.208) | (-0.746) |
|  | *6.8e-03* | *-.035* | *.019* | *.011* | *-1.0e-03* | *-3.1e-03* | *-3.4e-03* | *-.015* |
| Weight | 4.6e-03 | .02* | 8.1e-03 | -1.4e-03 | -1.6e-03 | .018* | -4.0e-03 | -6.9e-04 |
|  | (0.529) | (2.297) | (1.042) | (-0.184) | (-0.249) | (2.401) | (-0.512) | (-0.079) |
|  | *9.8e-03* | *.052* | *.019* | *-4.2e-03* | *-4.4e-03* | *.057* | *-9.4e-03* | *-1.8e-03* |
| Happiness | 1.9e-04 | -4.2e-04 | 8.7e-04 | 7.7e-04 | -8.0e-04† | -2.4e-03** | -4.0e-04 | 4.4e-04 |
|  | (0.290) | (-0.445) | (1.431) | (0.916) | (-1.650) | (-3.144) | (-0.687) | (0.480) |
|  | *5.2e-03* | *-.011* | *.027* | *.023* | *-.03* | *-.075* | *-.012* | *.011* |
| Health | -4.7e-03*** | -1.9e-03† | -6.8e-04 | -2.9e-04 | -1.6e-03* | -1.1e-03 | -1.3e-03† | -6.0e-04 |
|  | (-5.399) | (-1.905) | (-0.850) | (-0.319) | (-2.354) | (-1.318) | (-1.746) | (-0.625) |
|  | *-.099* | *-.043* | *-.016* | *-8.0e-03* | *-.046* | *-.032* | *-.032* | *-.014* |
| Education | .039*** | .058*** | .067*** | .07*** | -.073*** | -.024* | .1*** | .1*** |
|  | (4.504) | (4.632) | (8.565) | (6.518) | (-10.907) | (-2.342) | (14.218) | (8.938) |
|  | *.073* | *.099* | *.139* | *.143* | *-.182* | *-.051* | *.21* | *.183* |
| Income | .015* | .031** | 4.0e-04 | -4.8e-03 | 8.8e-03† | .057*** | .012* | -7.5e-03 |
|  | (2.374) | (2.927) | (0.073) | (-0.468) | (1.848) | (5.369) | (2.222) | (-0.687) |
|  | *.043* | *.066* | *1.3e-03* | *-.012* | *.036* | *.151* | *.04* | *-.017* |
| Marital Status: Single | 5.2e-03 | .095** | -.065** | -.026 | .019 | -6.6e-03 | -.021 | .04 |
|  | (0.190) | (2.832) | (-2.688) | (-0.904) | (0.963) | (-0.241) | (-0.860) | (1.257) |
|  | *3.2e-03* | *.057* | *-.044* | *-.019* | *.015* | *-4.9e-03* | *-.014* | *.025* |
| Have Offspring | -.044 | .016 | -.032 | -.12** | 2.9e-03 | .13*** | -.011 | 4.7e-03 |
|  | (-1.372) | (0.354) | (-1.096) | (-3.140) | (0.123) | (3.617) | (-0.400) | (0.110) |
|  | *-.027* | *9.3e-03* | *-.022* | *-.084* | *2.4e-03* | *.091* | *-7.5e-03* | *2.8e-03* |
| Heterosexual | .019 | .14*** | .045† | -.058* | -.12*** | 4.3e-03 | .071** | -.023 |
|  | (0.707) | (4.243) | (1.856) | (-2.119) | (-5.863) | (0.159) | (3.012) | (-0.746) |
|  | *.011* | *.084* | *.029* | *-.042* | *-.089* | *3.1e-03* | *.045* | *-.014* |
| Self-rated Attractiveness | -2.5e-04 | 1.1e-03 | 6.3e-04 | 1.4e-04 | -7.0e-04 | 1.1e-03 | 2.1e-03** | 1.2e-03 |
|  | (-0.293) | (1.074) | (0.822) | (0.156) | (-1.119) | (1.280) | (2.807) | (1.196) |
|  | *-5.4e-03* | *.025* | *.015* | *3.8e-03* | *-.021* | *.031* | *.05* | *.028* |
| Constant | .83*** | .67*** | .015 | -.28 | -1.3*** | -1.3*** | .25 | .55** |
|  | (4.411) | (3.320) | (0.089) | (-1.544) | (-9.158) | (-7.556) | (1.524) | (2.800) |
| Observations | 4116 | 2521 | 4106 | 2519 | 4106 | 2519 | 4106 | 2519 |
| *R*^2^ | 0.025 | 0.043 | 0.030 | 0.034 | 0.058 | 0.036 | 0.085 | 0.077 |
| F | 8.981 | 9.500 | 10.68 | 7.593 | 20.08 | 6.404 | 35.39 | 18.52 |

*Notes*: OLS regressions. *t*-statistics in parentheses (standard errors adjusted for heteroscedasticity). Standardized regression coefficients in italics. † *p* < .10; * *p* < .05; ** *p* < .01; *** *p* < .001.

**A9 Table.** Sex-specific effects on the importance of personality on sexual attraction.

|  | *Males* | *Females* | *Males* | *Females* | *Males* | *Females* | *Males* | *Females* |
| --- | --- | --- | --- | --- | --- | --- | --- | --- |
|  | (1) | (2) | (3) | (4) | (5) | (6) | (5) | (6) |
| *Dep. Var.* | Personality | | Trust | | Openness | | Emotional Connection | |
| Age | .03*** | .027* | .018* | 8.2e-03 | .03*** | .03*** | 8.4e-04 | .017† |
|  | (3.874) | (2.560) | (2.426) | (0.883) | (4.557) | (3.456) | (0.111) | (1.646) |
|  | *.449* | *.381* | *.279* | *.13* | *.522* | *.489* | *.013* | *.261* |
| Age*Age | -2.9e-04** | -2.4e-04† | -1.5e-04 | -5.2e-05 | -2.6e-04*** | -2.9e-04** | 6.3e-06 | -1.7e-04 |
|  | (-3.159) | (-1.753) | (-1.625) | (-0.429) | (-3.464) | (-2.661) | (0.071) | (-1.298) |
|  | *-.354* | *-.249* | *-.181* | *-.061* | *-.377* | *-.356* | *8.0e-03* | *-.195* |
| Height | -.036* | .014 | -.048** | 2.0e-03 | -.034* | -.022 | 1.6e-03 | -5.0e-03 |
|  | (-2.106) | (0.683) | (-2.849) | (0.114) | (-2.434) | (-1.304) | (0.091) | (-0.253) |
|  | *-.036* | *.014* | *-.049* | *2.3e-03* | *-.04* | *-.026* | *1.6e-03* | *-5.4e-03* |
| Weight | .028** | .013 | .026** | .017* | .03*** | .029*** | 4.8e-03 | -.011 |
|  | (3.255) | (1.488) | (3.105) | (2.143) | (4.126) | (3.903) | (0.566) | (-1.169) |
|  | *.061* | *.034* | *.057* | *.049* | *.075* | *.087* | *.011* | *-.029* |
| Happiness | 1.1e-03† | 2.8e-03** | 8.4e-04 | 1.8e-03* | -2.0e-04 | 2.1e-03* | 6.8e-04 | 8.8e-04 |
|  | (1.683) | (2.967) | (1.265) | (2.165) | (-0.359) | (2.567) | (1.094) | (1.028) |
|  | *.031* | *.07* | *.024* | *.05* | *-6.4e-03* | *.063* | *.02* | *.024* |
| Health | 1.5e-03† | 4.9e-04 | 8.0e-04 | 9.6e-04 | 4.5e-04 | 1.5e-03 | -2.4e-03** | -2.0e-03* |
|  | (1.712) | (0.487) | (0.942) | (1.020) | (0.607) | (1.636) | (-2.775) | (-2.082) |
|  | *.032* | *.011* | *.018* | *.025* | *.012* | *.04* | *-.053* | *-.049* |
| Education | -.015† | -1.3e-03 | -.034*** | -.039*** | -.018* | -.016 | 1.4e-03 | .029* |
|  | (-1.663) | (-0.112) | (-3.966) | (-3.727) | (-2.409) | (-1.642) | (0.161) | (2.397) |
|  | *-.027* | *-2.3e-03* | *-.065* | *-.077* | *-.039* | *-.033* | *2.7e-03* | *.054* |
| Income | -.025*** | -.047*** | -.019** | -.025** | -4.5e-03 | -.011 | -.013* | -.026* |
|  | (-4.120) | (-4.398) | (-3.186) | (-2.694) | (-0.934) | (-1.181) | (-2.078) | (-2.460) |
|  | *-.076* | *-.102* | *-.059* | *-.062* | *-.016* | *-.028* | *-.04* | *-.062* |
| Marital Status: Single | -.041 | -.12*** | -1.1e-03 | -.015 | -.071** | -.01 | .063* | -.09** |
|  | (-1.562) | (-3.509) | (-0.045) | (-0.518) | (-3.243) | (-0.364) | (2.437) | (-2.845) |
|  | *-.026* | *-.071* | *-7.2e-04* | *-.011* | *-.052* | *-7.4e-03* | *.041* | *-.059* |
| Have Offspring | .091** | -.028 | .057† | .054 | .061* | 3.5e-03 | .065* | -.048 |
|  | (2.874) | (-0.655) | (1.860) | (1.470) | (2.389) | (0.097) | (2.074) | (-1.186) |
|  | *.056* | *-.017* | *.036* | *.036* | *.045* | *2.4e-03* | *.041* | *-.031* |
| Heterosexual | -.088*** | -.22*** | -.034 | -.027 | -.14*** | -.25*** | .017 | .079* |
|  | (-3.294) | (-6.751) | (-1.271) | (-0.929) | (-6.105) | (-9.107) | (0.656) | (2.546) |
|  | *-.051* | *-.133* | *-.02* | *-.018* | *-.094* | *-.177* | *.011* | *.051* |
| Self-rated Attractiveness | -2.7e-03** | -4.9e-03*** | -3.8e-03*** | -5.0e-03*** | 1.2e-03 | -9.1e-04 | -1.5e-03† | -4.3e-03*** |
|  | (-3.208) | (-4.906) | (-4.682) | (-5.519) | (1.584) | (-1.001) | (-1.796) | (-4.375) |
|  | *-.06* | *-.114* | *-.085* | *-.132* | *.031* | *-.025* | *-.034* | *-.108* |
| Constant | -.55** | -.27 | .43* | .68*** | -.14 | -.26 | .48** | .58** |
|  | (-2.981) | (-1.322) | (2.395) | (3.776) | (-0.911) | (-1.526) | (2.643) | (2.886) |
| Observations | 4116 | 2521 | 4106 | 2519 | 4106 | 2519 | 4106 | 2519 |
| *R*^2^ | 0.037 | 0.054 | 0.038 | 0.040 | 0.063 | 0.069 | 0.010 | 0.026 |
| F | 13.21 | 12.11 | 13.02 | 8.777 | 21.09 | 16.06 | 3.665 | 5.498 |

*Notes*: OLS regressions. *t*-statistics in parentheses (standard errors adjusted for heteroscedasticity). Standardized regression coefficients in italics. † *p* < .10; * *p* < .05; ** *p* < .01; *** *p* < .001.
