# Supplementary material for: Sex differences in sexual attraction for aesthetics, resources and personality across age
Source: PLoS One. 2021 May 19;16(5):e0250151. doi: 10.1371/journal.pone.0250151 (PMC8133465; doi:10.1371/journal.pone.0250151)
Supplement: Attachment — Submitted filename: Respons_to_reviewers.docx [file pone.0250151.s002.docx]

**PONE-D-19-32550**
*The importance of sexiness: The impact of biological and socio-economic characteristics on human sexual attraction*

Dear Dr Jones,

We would like to sincerely thank yourself as editor and both reviewers for such a comprehensive and informative review. As you note in your review the large and diverse sample size offers the opportunity for a unique and significant contribution to the mate choice literature. That said we also acknowledge your comments requesting major revisions.

We have addressed all of the comments and incorporated all of the requested changes and edits, by completing a major re-write of the study. We have incorporated the majority of the new literature and theory suggested by the reviewers into the re-write. We have also conducted more and completely new analysis based on the reviewers and your feedback.

In line with both of the reviewers comments we have completely revised the entire analysis section (Reviewer 1), and slightly re-aligned the scope of the manuscript to provide a more nuanced paper on both absolute and relative sex differences across age (Reviewer 2), with rewrites to the abstract, introduction, background and discussion.

We again would like to express our gratitude for the opportunity to resubmit our revised manuscript to PLoS One.

Thank you,

**Response to Editors**

1. The claim that females remain unaffected by mating temporality is overstated. Females tend to prefer more masculine men facially for short term relationships (Jones et al, 2019; Psychological Science), and women seem to choose more muscular men for short term mating (Frederick & Haselton, 2007; Personality and Social Psychology Bulletin).
2. Reviewer 2 points out more of these kinds of overstatements, and this needs to be drastically changed.
3. There seem to be a lot of confusion around statistical terms and practice. For example, on page 12, line 7, you describe a set of multivariate analyses. Multivariate analysis involves a set of multiple Y and single-to-multiple X (in the context of regression) variables; whereas the data described here seems to simply reflect a large number of models, split by sex of participant, for age, attractiveness, build, etc (and the other kinds of ‘grouped’ variables). This not a multivariate analysis - though you certainly have scope to do so - and so should not be described as such.
4. The notes on the tables also claim these regressions are ‘robust OLS regressions’. It is not mentioned anywhere that these regressions are robust regression in the statistical sense of the word - indeed, robust regression penalises outliers through various methods and is therefore not ordinary least squares.
5. Reviewer 2 also asks whether any kind of multiple correction was done, and this should be reported if done - or carried out if so. Finally, the use of the Epanechnikov kernel function as a way to test relative preferences of a given trait seems too complex for what can be approximated with linear models - if there is a specific reason for using this method it should be stated and expanded upon in the methods section before it is introduced. The difference between female and male groupings at different age categories was also confusing.
6. In addition to addressing the queries of both reviewers, here is an analytical strategy that I think would make sense. The authors are free to reject this idea, but it is important to have a clear and concise analysis that allows readers to interpret what is going on in the data entirely, and without the overwhelming set of numbers in the manuscript as-is.
7. First, I suggest computing a principal components analysis or factor analysis of the ratings of the nine traits for the entire dataset (grouped here by Aesthetic, Resource and Personality). The labels assigned to these are fair groupings, but it is very unlikely that they will not be correlated in some fashion with one another - a simple idea would be age and income, or openness and intelligence, for example). A rigorous PCA or FA would boil this multidimensional data-space into something more coherent. You can then in a more data driven way provide a set of groupings by checking how each of the individual traits correlate with the new factors. An ideal setting perhaps would be finding 3 factors, wherein age, attractiveness, and physical build correlate strongly with the first factor, education, income, and intelligence with the second but not the first and so on - this may or may not occur, but the correlations are important.
8. Second, it is then possible to take the characteristics of your participants as you did in the later analyses (reported in the tables) and, in three models if you prefer, regress these demographics against the factors.
   If you find, say, a factor that captures attractiveness and build, perhaps you will find that the sex of the participant (now entered as a predictor) will associate more strongly with this factor - i.e. men care more about this constellation of traits represented by the factor than women do.
9. You can also test your interactions properly here in a correctly specified linear model, by allowing say age and sex to interact - do older men care less about this factor than women? This can be properly tested as at the moment the number of models is too much and they are isolated.

**Points 1-9** Editors comments–

We have addressed all of the comments and incorporated all of the requested changes and edits suggested by the editor. We have also conducted more robust and completely new analysis based on the editors and the reviewer’s helpful feedback. The manuscript has been almost completely rewritten in accordance with the suggested reviews.

**Response to Reviewers**

**Reviewer #1**

10. **Thank you for inviting me to review this paper. The study is a large sample’s report on the perceptions of attractive features in others. The topic is interesting and it is good to see large sample science.**

- The authors are appreciative of the positive feedback.

11. **Abstract
    I found the wording of the results in the introduction a little confusing. In particular, it is not made clear how an interaction analysis can reveal a sex difference effect (“our interaction analysis across age pinpoints a stronger male preference for attractiveness and physical build but a stronger female preference for intelligence, trust, and emotional connection.” This latter part appears to be the interaction: “Though of these sex differences show large variation or change at different life stages for both sexes”**

- The abstract has been completely rewritten in line with the reviewers comments..

***Introduction*
It would be good to cite more work critical of the good genes hypothesis. Research on health and behavioural outcomes of attractive physiology since Trivers (1972) and Scheib et al (1999) suggest that this is not as simple as is suggested in the opening background paragraph. Some of the key findings in good genes do not replicate (Thornhill & Gangestad, 2006; Foo, 2017). Most recently, Cai et al (**[**https://psyarxiv.com/hnbv7/**](https://urldefense.com/v3/__https:/psyarxiv.com/hnbv7/__;!!NVzLfOphnbDXSw!SR39sAA4oRuThnhWbqvNmgMzoPR-oR4mEtB4svdnM96MYBKIxDvGIb-le8FqOtMFOXA$)**) show this with health outcomes related to attractiveness, averageness, femininity and coloration. Whilst I would not expect a comprehensive review to be added (I am aware of space limitations), it would be good to see some caveats in the largely uncritical background.**

- The introduction has been completely rewritten in line with the reviewers comments.

12. **It would be useful and transparent to end the introduction with operationalised hypotheses. This would also add clarification as to why the selected ‘personality traits’ are used. (I appreciate some of this appears in the method section but given the theory-informed nature of this study hypotheses and predictions would be expected).**

- The introduction has been completely rewritten in line with the reviewers comments.

13. **It would useful to have a summary of the evidence of the relationship between self-reported attractiveness preferences and tested influence of attractive features. I am not familiar with evidence that the relationship between self-reported importance of, say intelligence, for attractiveness and the contribution that intelligence has in overall perceptions of attractiveness on meeting new people. This content would strengthen the justification for the paper’s core methodology.**

- The authors are not aware of any such literature demonstrating clear evidence as per the reviewers request, however the authors have rewritten the introduction section with greater theoretical justification.

14. **Method
    “Each question thus asks respondents to rank the level of importance they assign to each characteristic in relation to sexual attraction on a sliding scale from 1 to 100.” -> does this mean that the concrete activity was to produce a hierarchy (ordinal) data or was each attributed presented and intended to be evaluated in isolation?**

- The relative importance is ordinal by nature. However inline with the reviewers comments our study now incorporates both individual evaluations of importance per characteristic, but also now relative to other characteristics.

15. **Results
    Table 1 should include some effect size metrics, especially given the sample size. Perhaps even include metrics such as the overlap coefficient (see Inman and Bradley, 1989). For example, the largest difference between sexes on Physical build/features t=19.32 has an 81% overlap in distribution of responses which is informative for a reader for understanding what a difference between the sexes mean in a concrete sense.**

- We thank the reviewer for the suggestion. We have now calculated and shown the overlapping coefficients between each pair of distributions. In addition, we also note that the effect size can also be gauged for the relative difference in terms of mean comparison, as the unit () can be viewed as SD from individual’s mean

16. **The transparency of the table could be improved by clarifying ‘upper’ and ‘lower’ headings.**

- *We have now changed the them to 95% CI* Lower and 95% CI Upper, respectively.

17. **The explanation of figure 2 invites the reader to draw inference from their perceived difference in the difference in effect of linear prediction across age to understand sex differences in the sample. I would strongly advise against this. This would encourage the testing of an age*sex interaction to demonstrate this effect empirically.**

- Figure 2 has been removed in line with the reviewer’s comments.

18. **As far as I can tell, other than the mentions in the abstract and discussion there is no empirical evaluation of the interaction between sex and age in this study. Tables 2, 3 and 4 test the effects of age within the sexes but not across (using an interaction, Fisher’s z’ or Steiger’s T/z tests) the sexes. i.e. Tables 2, 3 and 4 column 1 conducts analysis on only the 4106 male participants and then column 2 conducts analysis on only the 2519 female participants. Therefore, there are concerns about making comments on the observed difference in the relationships displayed by the sexes. The authors refer to interaction analysis**

- The authors have completely rewritten the method and analysis sections in line with the reviewers comments.

19. **I strongly recommend that the authors consider including sex*age interaction effects on the traits or they report tests of the size of different effects (i.e. Steiger’s T).**

- The authors have completely rewritten the method and analysis sections in line with the reviewers comments.

20. **The authors do refer to interactions throughout but no interaction tests appear in this version of the manuscript.**

- The authors have completely rewritten the method and analysis sections in line with the reviewers comments.

21. **Discussion
    The authors conclude “More specifically, our interaction analysis of sex differences in preferences across age…” however I am not able to find any interaction tests in the current paper.**
22. **The authors note at the end of their discussion “Admittedly, however, in cyber mating market settings, stated preferences are not always a good indicator of actual behaviour”. This should have much more prominence in the discussion (and perhaps introduction) as it caveats the implications of the research significantly.**

- This point has been rewritten, as has all of the discussion section.

23. **Overall this paper adds a general description of sex differences in self-reported points of attraction to the literature. There needs to be inferential tests of the sex*age interaction and the paper’s final caveat should be much more prominent as the study is reliant on the honesty of those engaging with “cyber mating markets”**

- This point has been rewritten, as has all of the discussion section.

**Reviewer #2:**

24. **This review uses a large dataset from Australia to examine sex differences in mating preferences for traits related to physical attractiveness, the ability to provide resources, and three different facets of personality important for pair bonding.**

- The authors are appreciative of the positive feedback.

25. **There are numerous merits to the article. It uses models which account for linear and curvilinear relationships with age, which gives an indication of lifetime trajectories of mating preferences (and their associated sex differences). This is particularly relevant given that a considerable amount of mate preference research is conducted on young adults. Should sex differences in preferences changes across the life span, then conclusions drawn from young adults alone may exaggerate or understate sex differences.**

- The authors are appreciative of the positive feedback.

26. **The methodology of the paper and the chosen forms of analysis are sound. And the results could make a decent theoretical contribution to the area. However, the paper has some weaknesses that make it unsuitable for publication in its current form.**

- The authors are appreciative of the positive feedback.

27. **The main weakness is that it presents many data, but does very little in the way of actually interpreting the results. That is, it is too descriptive, and the precise merits of looking at the changes in mate preferences across age is missing from the narrative. The primary analysis of the study focuses on the possible non-linear relationship of mate preferences and age. Historically, the majority of mate choice research has focussed on those in the age group of peak fertility participants, primarily utilising young adult (student) samples. The novelty of the current studies broader age distribution and sample size allows the researchers to potentially plot lifetime trajectories of mating preferences and their associated sex differences.**

- The authors are appreciative of the positive feedback. We have incorporated the reviewers comments into both the method and discussions sections of the rewrite.

28. **A second weakness is the lack of specific details surrounding the choice of the statistics and presentation methods used, which makes the benefits of this approach relative to others hard to decipher. I recommend that the article be revised and resubmitted, with substantially expanded results and discussion sections, and a focus on the novel contributions to knowledge this analysis provides. Below, I have given some more specific recommendations that I hope may improve the paper. Those which I consider to be major issues, I have marked with an asterisks**.

- The authors are appreciative of the positive feedback. We have incorporated the reviewers comments into both the method and discussions sections of the rewrite.

29. **Abstract: I find it odd that t-tests are mentioned here rather than the OLS regression and kernel-smoothing plots, which I think give a much more nuanced interpretation of the data.**

- The authors are appreciative of the positive feedback. We have incorporated the reviewers comments into the method, analysis and discussions sections of the rewrite.

30. **Page 3, line 102. It is worth considering that the good genes hypothesis isn’t the only explanation for the preference for physical attractiveness. In fact the sexy sons hypothesis (or should we say, sexy offspring, in paid bonded species) appears to be a stronger force for selection. See a recent meta-analysis of 55 species by Prokop et al. (2012).**

- The authors have incorporated broader literature into both the introduction and discussion, moving the paper towards more of an exploration of relative sex differences.

31. ***Page 4, line 117. The preceding paragraph polarises the sex differences between the sexes by applying the logic of MCFC (Males compete, females choose) species to humans which have mutual mate choice. There is a large sex differences in obligate parental investment in humans, but sex differences in *typical* parental investment is much smaller, leading to similar choosiness in the long-term mating domain of humans. Humans are a MMC (mutual mate choice) species, and sex differences are relative, rather than absolute. Furthermore, polarising sex differences can be detrimental to scientific communication and pedagogy (see Stewart-Williams and Thomas, 2013). I urge the authors to acknowledge the reduced sex differences in pair-bonded species like humans by tempering their narrative here.**

- The authors appreciate the reviewers feedback and have incorporated the suggested literature into both the introduction and discussion, moving the paper towards more of an exploration of relative sex differences.

32. **Page 4, line 120-3. As above, physical attractiveness is *relatively* more important for men, but is still absolutely important for both sexes. And this effect appears to be present in different cultures, even when mate preferences are constrained: see Thomas et al., (2019) for a recent study looking at samples from the UK, Norway, Australia, Singapore, and Malaysia.**

- The authors appreciate the reviewers feedback and have incorporated the suggested literature into both the introduction and discussion, moving the paper towards more of an exploration of relative sex differences.

33. **Page 4, line 133-4. What does this study add that is not already known? This question has been examined countless times since the Second World War, with an additional focus on cross-cultural differences since the late 1980’s. There is a unique contribution here that I think is important, but the authors are expecting the reader to go out of their way to figure out what it is.**

- The authors have substantially rewritten the manuscript, and provided clear justification, as well as analysis and findings for the reader.

34. **Page 5, line 140. The physical health = attractiveness connection is not as clear as initially thought (for a meta-analysis see Weeden & Sabini, 2005). More likely, men’s increase preference for physical attractiveness relates to its association with youth and, therefore, fecundity. Page 6, lines 169-170. Are these economic factors the only driving force behind these preferences? It seems odd that the previous section relies on evolutionary arguments, but this one considers the driver for resource provision as the consequence of modern day market forces – especially seeing as provision for neonatal offspring is one of the most clear-cut selection pressures faced by humans throughout our history.**

- The authors have substantially rewritten the manuscript, and provided clear justification, as well as analysis and findings for the reader.

35. **Page 6, lines 179-181. Is this really the case? Thomas et al., (2019), for example, found that culturally disparate groups, with different views on sex discrimination and egalitarianism, showed remarkable consistency in their preference for one of the personality traits key in successful pair bonding: kindness.**

- The authors appreciate the reviewers feedback and have incorporated the suggested literature into the introduction, moving the paper towards more of an exploration of relative sex differences.

36. ***Page 7, line 195. The fact that relationship context is not given, and that relationship preferences vary across short- and long-term domains (see Stewart-Williams et al., 2017; Thomas and Stewart-Williams, 2018; and Thomas, 2018) is problematic. One assumes that the participants are answering in line with their sociosexuality so that those who want short-term mates answer in one context, and those who want long-term mates answer in the others. This needs to be addressed in the discussion.**

- The authors appreciate the reviewers feedback and have incorporated the suggested literature into the introduction rewrite

37. **Page 8, line 220. What is the reason for men to outnumber women 1.7 to 1? Seems unusual for a national survey.**

- Data was collected via survey of online dating participants. Such a male dominated sex ration is arguably normal in this setting.

38. **Table 1. Were any corrections made for multiple comparisons?**

- Table 1 has been removed.

39. **Page 9, lines 242-7. More clarity is needed regarding the selection of the Epanechinikov kernel function**.

- Greater methodological justification has been prodided in each Figure.

40. **Figure 2. The notes should include which group the red reference line refers to.**

- Table 1 has been removed.

41. **Page 12, lines 7+. The multivariate analysis section. This whole section talks about which betas are significant and in what direction, but has very little in the way of interpretation of the results. It may be that the authors prefer to do this in the discussion section, but it is absent from there as well. The three tables present many data, and a more nuanced discussion of directions of findings, strengths of findings, and within category consistency and deviation is appropriate. There is also very little discussion of the age effects, and what implications a curvilinear pattern has for how we view sex differences.**

- We have conducted new and more robust analysis based on the editors and the reviewer’s helpful feedback.

  References

  Prokop, Z. M., Michalczyk, Ł., Drobniak, S. M., Herdegen, M., & Radwan, J. (2012). Meta‐analysis suggests choosy females get sexy sons more than “good genes”. Evolution: International Journal of Organic Evolution, 66(9), 2665-2673.

  Stewart-Williams, S., & Thomas, A. G. (2013). The ape that thought it was a peacock: Does evolutionary psychology exaggerate human sex differences?. Psychological Inquiry, 24(3), 137-168.

  Stewart-Williams, S., Butler, C. A., & Thomas, A. G. (2017). Sexual history and present attractiveness: People want a mate with a bit of a past, but not too much. The Journal of Sex Research, 54(9), 1097-1105.

  Thomas, A. G., & Stewart-Williams, S. (2018). Mating strategy flexibility in the laboratory: Preferences for long-and short-term mating change in response to evolutionarily relevant variables. Evolution and Human Behavior, 39(1), 82-93.

  Thomas, A. G. (2018). Lowering partner standards in a short‐term mating context. In T. K. Shackelford, & V. A. Weekes‐Shackelford (Eds.), Encyclopedia of evolutionary psychological science (pp. 1– 3). Cham, Switzerland: Springer International Publishing.

  Thomas, A. G., Jonason, P. K., Blackburn, J. D., Kennair, L. E. O., Lowe, R., Malouff, J., ... & Li, N. P. (2019). Mate preference priorities in the East and West: A cross‐cultural test of the mate preference priority model. Journal of personality.

Weeden, J., & Sabini, J. (2005). Physical attractiveness and health in Western societies: a review. Psychological bulletin, 131(5), 635.
